# A mitogenomic perspective on the ancient, rapid radiation in the Galliformes with an emphasis on the Phasianidae

**DOI:** 10.1186/1471-2148-10-132

**Published:** 2010-05-06

**Authors:** Yong-Yi Shen, Lu Liang, Yan-Bo Sun, Bi-Song Yue, Xiao-Jun Yang, Robert W Murphy, Ya-Ping Zhang

**Affiliations:** 1 State Key Laboratory of Genetic Resources and Evolution, Kunming Institute of Zoology, the Chinese Academy of Sciences, Kunming 650223, China; 2 Laboratory for Conservation and Utilization of Bio-resources, Yunnan University, Kunming 650091, China; 3 Graduate School of the Chinese Academy of Sciences, Beijing 100000, China; 4 Sichuan Key Laboratory of Conservation Biology on Endangered Wildlife, College of Life Science, Sichuan University, Chengdu, Sichuan 610064, China; 5 Department of Natural History, Royal Ontario Museum, 100 Queen's Park, Toronto, Ontario M5S 2C6, Canada

## Abstract

**Background:**

The Galliformes is a well-known and widely distributed Order in Aves. The phylogenetic relationships of galliform birds, especially the turkeys, grouse, chickens, quails, and pheasants, have been studied intensively, likely because of their close association with humans. Despite extensive studies, convergent morphological evolution and rapid radiation have resulted in conflicting hypotheses of phylogenetic relationships. Many internal nodes have remained ambiguous.

**Results:**

We analyzed the complete mitochondrial (mt) genomes from 34 galliform species, including 14 new mt genomes and 20 published mt genomes, and obtained a single, robust tree. Most of the internal branches were relatively short and the terminal branches long suggesting an ancient, rapid radiation. The Megapodiidae formed the sister group to all other galliforms, followed in sequence by the Cracidae, Odontophoridae and Numididae. The remaining clade included the Phasianidae, Tetraonidae and Meleagrididae. The genus *Arborophila *was the sister group of the remaining taxa followed by *Polyplectron*. This was followed by two major clades: ((((*Gallus*, *Bambusicola*) *Francolinus*) (*Coturnix*, *Alectoris*)) *Pavo*) and (((((((*Chrysolophus*, *Phasianus*) *Lophura*) *Syrmaticus*) *Perdix*) *Pucrasia*) (*Meleagris*, *Bonasa*)) ((*Lophophorus*, *Tetraophasis*) *Tragopan*))).

**Conclusions:**

The traditional hypothesis of monophyletic lineages of pheasants, partridges, peafowls and tragopans was not supported in this study. Mitogenomic analyses recovered robust phylogenetic relationships and suggested that the Galliformes formed a model group for the study of morphological and behavioral evolution.

## Background

The Galliformes, a well-known and widely distributed Order in Aves, contains about 70 genera and more than 250 species including the domestic chicken (*Gallus gallus*), green peacock (*Pavo muticus*) and turkey (*Meleagris gallopavo*), among others. Many galliforms have beautiful ornamentations and they play an important role in hunting and entertainment. Regardless, these birds are best known for their importance in agriculture and as model organisms in scientific studies [1-6].

**Figure 1:**
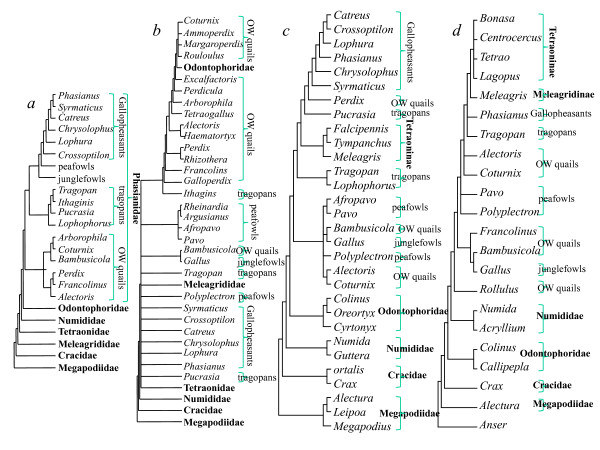
**Phylogenetic hypotheses from various molecular, morphological and behavioral analyses of gamebirds**. (a) The traditional classification from Johnsgard (1986); (b) morphological and behavioral data (Dyke et al., 2003); (c) combined data including two mitochondrial genes (*CytB*, *ND2*) and four nuclear introns (*BFib7*, *DCoH3*, *OvoG*, and *Rhod1*) (Kimball and Braun 2008); (d) insertion events of CR1 retrotransposable elements (Kaiser et al., 2007).

The phylogenetic relationships of galliforms have long been the focus of research [7-21]. Traditionally, the Galliformes contained seven families: Megapodiidae (scrubfowl and brush-turkeys), Cracidae (curassows and guans), Tetraonidae (grouse), Meleagrididae (turkey), Numididae (guineafowls), Odontophoridae (New World quails) and Phasianidae (pheasants and Old World quails). Phasianids formed the most diverse and complex group, including two lineages: the pheasants and Old World partridges (OW quails). The pheasants were further divided into four lineages: peafowls, gallopheasants, junglefowls and tragopans [22] (Fig. 1a). However, this taxonomy was not supported by recent molecular and morphological analyses (Fig. 1b, c, d), which failed to obtain a consistent result and added more fuel to an already heated debate.

Mitochondrial DNA analyses revealed that *Gallus *(Tribe Phasianini), *Francolinus *(Tribe Perdicini) and *Bambusicola *(Tribe Perdicni) clustered together, suggesting that pheasants and partridges were not monophyletic groups [9,12]. Subsequently, these findings were supported by the analyses of nuclear gene sequences [11], combined mt genes and nuclear gene sequences (Fig. 1c) [17,20] and sequences from retrotransposable elements (Fig. 1d) [19]. However, one morphological study [15] conflicted with these assessments (Fig. 1b). The phylogenetic relationships within the Galliformes, and especially the unsolved branching order in the Phasianidae, limited interpretations of their morphological and ecological convergent evolution. The absence of a phylogeny also impacted on conservation initiatives.

Internal nodes resolved by previous studies were generally very short. Further, bootstrap support values were very low and many species had unresolved relationships, suggesting a rapid radiation of the Phasianidae [8,9]. Although the resolution of branching orders during a rapid radiation has proven to be challenging, large DNA sequence datasets have a much higher probability of recovering a robust tree [23-27].

Recently, mt genomes have been widely used to reconstruct intractable phylogenies [28-33]. In general, mtDNA accumulates mutations at a relatively faster rate than nuclear DNA, thus making it particularly useful for revealing closely spaced branching events. Considering that previous phylogenetic studies based on a single gene or a few genes failed to resolve the internal branching orders within the Galliformes, we sequenced the complete mt genomes of 14 galliform birds, and obtained other mt genomes from GenBank (Additional file 1). Consequently, we used extensive mt genomes from major groups of galliform birds to infer their phylogenetic relationships.

## Results

### Characteristics of the mitochondrial genome

The general characteristics of mt genomes of 34 galliforms and six anserforms are summarized in Additional file 1. The lengths of the complete mt genomes range from 16,604 to 16,870 bp. Length differences are mainly due to variation in the Control Region (CR). The overall average nucleotide composition was A = 29.3%, C = 30.65%, G = 14.51% and T = 25.45%. All mitochondrial gene organizations conformed to the standard avian gene order (chicken) [31,34].

### Phylogenetic relationships

Tests for stationarity of base composition among the taxa for each mt gene revealed that the Galliformes did not differ significantly in their base content (*P *> 0.05). Only *Alectoris chukar *for *CoxI *and *ND5*, *Alectoris lathami *for *ND5*, and *Bonasa bonasia *for *CoxII*, *ND2 *and *ND4 *differed marginally significantly. However, all five outgroup taxa failed the stationarity tests for some data partitions as follows: *Anas platyrhynchos *for *CoxI*, CR, *ND2 *and *ND5*; *Anser albifrons *for CR, *ND2 *and *ND5*; *Anseranas semipalmata *for CR; *Aythya americana *for *CoxI*, CR, *ND2 *and *ND5*; *Branta canadensis *for *CoxI*, CR and *ND5*; and *Cygnus columbianus *for CR (Additional file 2). In summary, CR had the greatest number of cases (in all five outgroups) that deviated from stationarity, and *Anas platyrhynchos *and *Aythya americana *deviated from stationarity most frequently (four times).

Because we mainly used the combined sequences to reconstruct the tree, we also calculated base composition for the 12 protein-coding gene set (Additional file 3). We found that base composition was not significantly different in codon positions 1 and 2 among Galliformes, while for codon position 3, some species departed from the average composition.

The combined dataset of 12 protein-coding genes (10,886 aligned sites) revealed a single, robust tree using MP and ML, and BI produced a summary of numerous trees (Additional file 4) for the Galliformes. The Megapodiidae was the sister group of the remaining taxa, followed by the Numididae. The remaining clade formed a complex mixture involving the families Tetraonidae, Meleagrididae and Phasianidae. The Phasianidae contained seven lineages, each with very high BSPs as follows: Group 1 contained *Arborophila*; Group 2 included *Tragopan*, *Lophophorus *and *Tetraophasis*; Group 3 was composed of *Chrysolophus*, *Phasianus*, *Lophura*, and *Syrmaticus*; Group 4 contained *Perdix*; Group 5 had *Pucrasia*; Group 6 held *Gallus*, *Bambusicola *and *Francolinus*; and Group 7 contained *Coturnix *and *Alectoris*. A clade containing families Tetraonidae and Meleagrididae branched off from within the Phasianidae. Only the phylogenetic relationships of *Pavo *and *Polyplectron *remained unresolved (Additional file 4).

In order to investigate the possibility of a bias owing to substitution saturation, we plotted transitions and transversions against pairwise sequence divergence using 12 mt protein-coding genes (Additional file 5). Codon positions 1 and 2 were not saturated. Codon position 3 was saturated for transitions in the comparison between the Galliformes and outgroups. In addition, some species departed from the average base composition in codon position 3 (Additional file 3). In order to reduce the possible influence of these two biases on codon position 3, we implemented P12 and RY-coding methods. The resulting tree topologies were identical to those of the unweighted schemes. As expected, relatively lower bootstraps values were obtained (Additional file 6). Better resolution and higher BSPs were obtained from DNA datasets that included all substitutions, as opposed to those subjected to weighting.

Both of the combined RNA datasets (tRNAs and rRNAs) obtained less resolution compared to protein-coding genes (Additional file 7 and Additional file 8). The aligned rRNA sequences (combined *12S *and *16S *rRNA genes) were 2,744 aligned sites in length. The tRNA dataset (combined 22 tRNA genes) contained 1,612 bp of aligned sites. The individual partitions (13 protein-coding genes, *12S *rRNA, *16S *rRNA and *CR*) showed very limited power for phylogenetic inference, leaving many unresolved nodes (Additional file 9).

**Figure 2:**
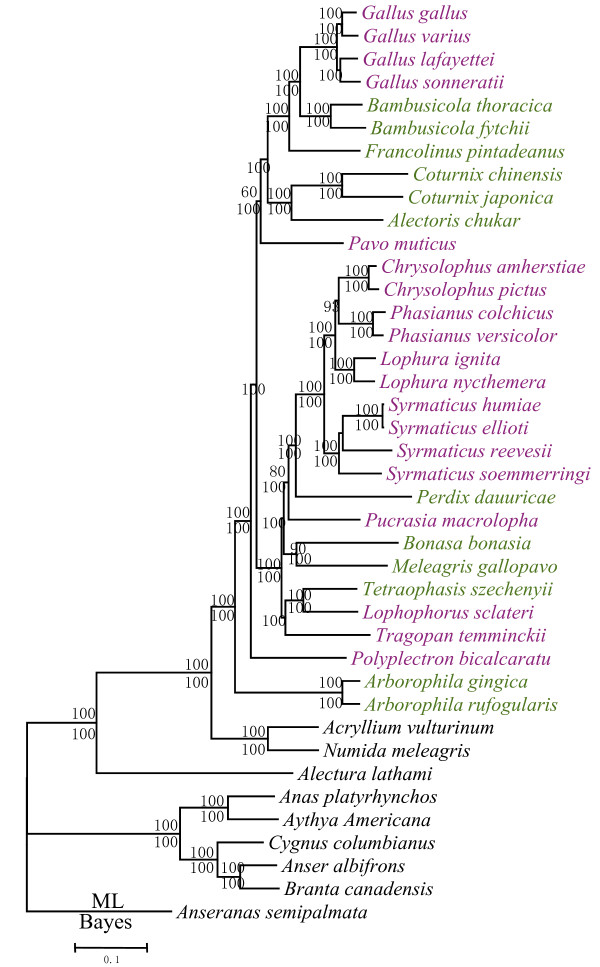
**Phylogenetic tree based on the complete mitochondrial genomes of galliform birds**. Bayesian posterior probabilities >70%, and maximum likelihood bootstrap proportions >50% are indicated on the branches. Species belonging to the Tribe Phasianini are marked in purple, and to Tribe Perdicini in green.

To maximize the amount of phylogenetic information, we pooled all mt genes (protein-coding, RNA and CR) to form a single dataset with a length of 16,508 aligned sites for a genomic-level phylogeny [33]. The BI analysis yielded a topology identical to that produced by the combined 12 protein-coding genes, except for the phylogenetic position of *Polyplectron*, which did not cluster with the peafowl, but rooted at the base of the Phasianidae, implying non-monophyly of peafowl (Fig. 2).

Regarding the phylogenetic positions of the Cracidae and Odontophoridae, the BI trees based on 10,502 nucleotide positions (Additional file 10) and 3,262 positions (Additional file 10) were very similar to those attained from complete mt genomes (Fig. 2). As expected, some nodes received relatively low statistical support.

### Assessing the performance of individual genes

**Figure 3:**
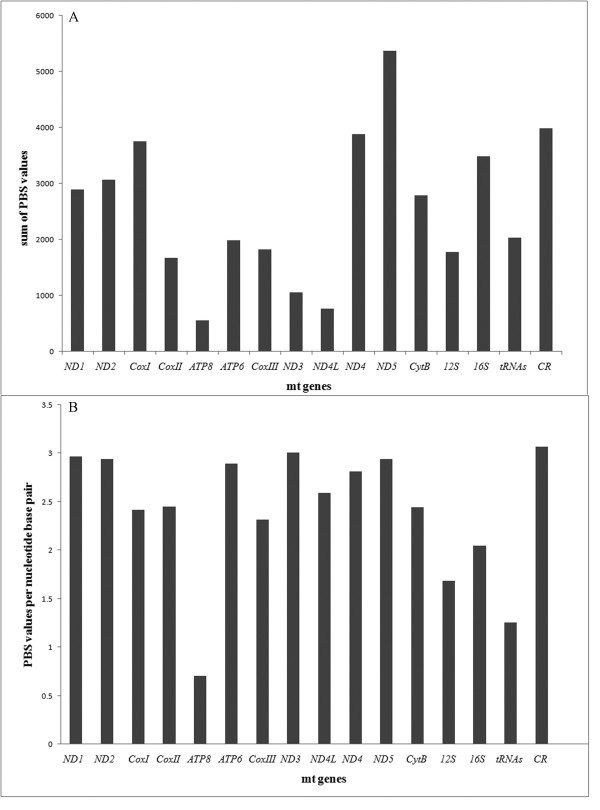
**Results of partitioned Bremer support (PBS) analyses with respect to each node on the mitochondrial genome tree**.

PBS analyses were performed to better understand the contribution of different parts of the mt genome on the genome phylogeny. Among the 16 partitions examined, *ND5 *provided the greatest contribution to tree resolution, followed by CR, while *ATP8 *contributed the least (Fig. 3A). In the analyses of PBS values per nucleotide base pair, CR performed the best and *ATP8 *was the worst (Fig. 3B).

## Discussion

The rapid radiation and convergent morphological evolution has confounded the resolution of relationships for many pheasants and partridges. Most previous molecular studies analyzed either one or a few mt genes [9,12,13,18] only or a single nuclear gene [11,35]. The paucity of data rendered the sequence of cladogenic events, especially within the family Phasianidae, unsolved. In this study, we showed that the number of genes, and thus the quantity of data, proved critical to resolving relationships. Individual genes had limited power to resolve the phylogeny of this group (Additional file 9). In contrast, the complete mt genomes clearly resolved most of the branching order within the pheasants and partridges, with strong nodal support.

At the base of the tree, *Alectura lathami *was the sister taxon of all other galliforms, followed in sequence by the cracids, new world quails and guineafowls, and then a clade including the Phasianidae, the Tetraonidae and the Meleagrididae (Additional file 10). This branching order was the same as obtained by some studies [9,12,18], yet other studies switched the positions of new world quails and guineafowl [13,17,20,36]. Unlike most previous studies based on morphology [15], a single nuclear gene [11], single mitochondrial gene [9], or retrotransposable elements [19], we obtained clear branching orders within the Phasianidae. *Arborophila *was the sister group to all other phasianids plus the Meleagrididae and Tetraonidae. Whereas the evolutionary relationships of *Perdix *remained unresolved in previous studies, in our study it clustered with the gallopheasants, and with strong nodal support (Fig. 2; 100% BSP and 1.00 BPP). *Tetraophasis *clustered independently with *Lophophorus *and their sister group was *Tragopan*; this association also enjoyed very high nodal support. And *Arborophila *did not cluster with other partridges. Therefore, the non-monophyly of the pheasants and partridges was more common than not, including the strongly supported association of *Gallus*, *Bambusicola *and *Francolinus*. *Polyplectron *did not cluster with *Pavo*, and *Pucrasia *did not cluster with other tragopans. These associations revealed that the peafowl and tragopan lineages were not monophyletic.

Our study and that of Kimball and Braun 2008 (Fig. 1c), based on combined data including two mt genes (*CytB*, *ND2*) and four nuclear introns (*BFib7*, *DCoH3*, *OvoG*, and *Rhod1*), were similar in some respects, but have many differences. For example, within *Gallus*, they resolved the species relationships as (((*G. lafayetti*, *G. varius*) *G. gallus*) *G. sonneratti*) and our ML and BI analyses resolved them as ((*G. gallus*, *G. varius*) (*G. lafayetti*, *G. sonneratti*)). Whereas no nodal support was obtained in the former set of relationships, our ML and BI trees were strongly supported at each node (Fig. 2; 100% BSP and 1.00 BPP). Kimball and Braun placed *Polyplectron *as the sister group to *Gallus *and *Bambusicola *but with less than 50% support values, yet all of our analyses located *Polyplectron *near the base of the clade for the Phasianidae (Fig. 2; 100% BSP and 1.00 BPP). Other discrepancies also occurred, such as the position of *Pavo*, *Perdix *and *Pucrasia*, and all of these differences received high support in our study.

Our study resolved many differences compared to trees based on retrotransposable elements (Fig. 1d) [19,36]. These studies placed *Gallus *near the base of the clade for the Phasianidae, followed by *Pavo*, and the remaining taxa split into two groups: (1) *Coturnix *and (2) gallopheasants, tragopans, turkey and grouse. In contrast, our analyses did not place *Gallus *near the base of the Phasianidae, but rather clustered it with *Coturnix*. This pair then clustered with *Pavo*, which then became the sister group of gallopheasants, tragopans, turkey and grouse (Fig. 2). Although Kriegs et al. (2007) did not resolve the position of the tragopans, we found them to be the sister group of the gallopheasants, and with very high support (Fig. 2; 100% BSP and 1.00 BPP). Studies of retrotransposable elements resolved the branching orders of seven families of Galliformes, however, many unsolved parallel relationships remained in the Phasianidae (Fig. 1d). Thus, retrotransposable elements were good markers to resolve relationships at the hierarchical levels of family, but seemed to be less powerful at resolving detailed relationships at the hierarchical levels of genus and species. In contrast, mt genomes were very informative at the level of genus/species, due to their relatively rapid rate of mutation.

Relative branch lengths suggest this group has undergone ancient, rapid radiations. Branching order is difficult to resolve during rapid radiations because of insufficient time for numerous genetic and morphological changes to accumulate [9]. Clearly, our combined mt genomes provide a greater abundance of information and thus may have a greater likelihood of fully resolving a tree than individual protein-coding genes and other subsets of mt genes (tRNA, and rRNA).

Because sequencing the complete mt genomes is expensive in both time and resources, the relative performance of individual genes is of great interest. Ranking individual genes by their respective contribution to the total PBS values--a rough indicator of phylogenetic utility--reveals that some genes, such as *ND5*, CR, *ND4, CoxI *and *16S*, are better indicators of galliform evolution than others (Fig. 3A). Longer genes tend to have more informative sites, and thus a larger total PBS. PBS value per nucleotide base pair can be used to maximize tree-acquisition efficiency. For galliform birds, *ND3*, *ND1*, *ND2*, *ND5*, *ATP6*, and *ND4 *are more informative than other mt genes (Fig. 3B). PBS values can be used with gene length, ease of amplification and sequencing to select a suite of genes for phylogenetic inference, especially given that different suites of genes may provide more information as a function of the tempo of evolution.

## Conclusions

Our robust mitogenomic tree indicates that galliform relationships are very complex. The traditional hypothesis of monophyletic lineages of pheasants, partridges, peafowls and tragopans is not supported in this study. Mitogenomics is a powerful tool for resolving the phylogenetic relationships of the Galliformes. Individual mitochondrial genes and nuclear genes seem to be less powerful in resolving phylogenetic relationships within the Galliformes, especially the Phasianidae. Clearly, complete mt genomes can provide more information, and thus are more powerful arbitrators of ambiguous phylogenetic relationships than partial genomes. Therefore, this strategy should serve to further resolve the phylogeny of the Galliformes as more mt genomes are obtained. Given the diversity of species in the Galliformes, and these birds' great complexity in morphological and behavior characters, such as flight, polygamy and sexual dimorphism, this Order can serve as an ideal model for a detailed study of character evolution. Thus, our study not only hypothesizes an evolutionary history of the Galliformes, it also provides primary genetic data for future studies. A caveat of this study is that, our tree tells us a part of story due to the only matrilineal heredity of mtDNA. In particular, our topology conflicts with that derived from nuclear DNA (nuDNA) retrotransposable elements [19,36]. These conflicts may be due to the different modes of heredity, and different tempos of evolution of mtDNA and nuDNA. Further study is required to resolve the current conflicts.

## Methods

### Specimens sampling

Muscle or feather tissue was obtained from 14 species. Further, 26 additional complete mt genomes were obtained from GenBank for the Galliformes and Anserformes (Additional file 1). No complete mt genomes were available for representatives of the Cracidae and Odontophoridae. Consequently, we mined GenBank and created a dataset with as many mitochondrial genes as possible for these taxa (Additional file 11).

### DNA extraction, PCR amplification, and sequencing

Total genomic DNA was extracted using standard 3-step phenol/chloroform extraction methods [37]. LA-PCR primer sets and segmental amplification primer sets were described previously [38]. An additional 96 species-specific primers were designed (Additional file 12).

LA-PCR amplifications were conducted using the following parameters: initial denaturation at 95°C 4 min, followed by 30 cycles of denaturation at 94°C for 30 sec, 58°C annealing extension for 16 min, and with a final extension at 72°C for 5 min. Subsequently, the LA-PCR products were used for segmental PCR amplification. PCR amplifications were conducted in a 50 μl volume containing 5 μl of 10 × reaction buffer, 0.2 mM dNTPs, 0.2 μM each primer, 1.5 U Taq DNA polymerase (TaKaRa Biosystems), and approximately 10 ng LA-PCR products. PCR amplifications were carried out using the following parameters: 95°C 4 min, 20 cycles of denaturation at 94°C for 1 min, annealing at 60-50°C (1 min; 0.5°C/cycle), extension at 72°C for 1 min, and finally 15 cycles of 94°C 1 min, 50°C 1 min, 72°C 1 min. PCR products were cleaned using Watson RCR Purification Kits (Watson BioTechnologies, Shanghai).

PCR products were sequenced at least three times in both directions on an ABI 3730 Sequencer (Applied Biosystems, Foster, CA, USA) using the ABI PRISM BigDye Terminator v3.0 sequencing kit. DNA sequences were edited using DNAstar Seqman software (DNASTAR Inc., Madison, WI, USA). The newly determined genomes were deposited in GenBank (GenBank accession numbers: FJ752423-FJ752436).

### Phylogenetic reconstruction

The sequence data were initially aligned using ClustalX 1.81 [39] with default parameters. Subsequently, the alignment was adjusted manually.

Because compositional bias among species can interfere with tree topology [40-42], prior to phylogenetic reconstruction, we performed tests of stationarity of base composition in TREEPUZZLE 5.2 [43]. Each gene and gene set was tested separately.

The combined sequence datasets of all 12 light-strand-encoded protein coding genes, two rRNA genes and 22 tRNA genes were analyzed separately using maximum likelihood and maximum parsimony (MP) implemented in PAUP* 4.0b10 [44]. MP heuristic searches used tree bisection reconnection (TBR) branch swapping executed for 10000 replicates. Modeltest 3.7 [45] was used to select the preferred models of evolution for ML, under the Akaike Information Criterion [46]. The GTR+I+G model was selected for the 12 coding genes and 22 tRNAs, and the GTR+G model had the best fit for the two rRNA genes. ML heuristic searches used TBR branch swapping executed in 10 replicates with the selected models. Because heuristic searches in PAUP* are very slow, we used two additional fast ML-based inference packages using 1 000 replicates each: RAxML [47] and PHYML [48]. Because their topologies are the same, and only a few bootstrap values are slightly different, we only present trees with bootstrap values from PAUP*.

Bayesian inference (BI) was performed using MrBayes 3.1.2 [49]. The Bayesian posterior probabilities (BPP) used models estimated with Modeltest 3.7 under AIC. Two separate runs were performed with four Markov chains. Each run was conducted with 5 × 10^6 ^generations and sampled every 100 generations. When the log-likelihood scores were found to stabilize, a consensus tree was calculated after omitting the first 25% trees as burn-in.

In order to detect the possible bias of substitution saturation, we plotted transitions and transversions against the pairwise sequence divergence using 12 mitochondrial protein-coding genes in DAMBE [50].

Two additional weighting strategies were applied in the analysis of combined 12 protein-coding genes to avoid possible bias of nucleotide composition and saturation: (1) excluding the 3rd codon positions, and (2) recoding the 3rd codon position nucleotides to two-state categories, R (purine) and Y (pyrimidine), i.e., RY-coding. RY-coding can greatly improve consistency in phylogenetic resolution by reducing bias from differences in nucleotide composition [51,52].

To examine the performance of individual genes, a partitioned Bremer support (PBS) analyses [53] were performed. The 13 protein-coding genes, 12S rRNA, 16S rRNA and control region (*CR*) partitions were each used to reconstruct the phylogeny using BI.

To maximize the amount of phylogenetic information, the entire mt genome (13 protein-coding genes, two rRNA genes, 22 tRNA genes and *CR*) was also used to reconstruct the phylogeny using BI, ML and BP methods.

Complete mt genomes were not available for representatives of the Cracidae and Odontophoridae. Thus, we mined GenBank to attain as many mt genes as possible for 11 species in the Cracidae and four in the Odontophoridae (Additional file 11). We combined these data with those from the complete mt genomes. Given the tradeoff between alignment length and taxonomic coverage, we compiled a dataset of 10502 nucleotide sites for 11 species in the Cracidae and 40 in the Galloanserae that have complete mt genomes. In addition, a dataset of 3262 bp was compiled for 11 representatives of the Cracidae, four Odontophoridae, and 40 Galloanserae. In these two datasets, substitution models were estimated with Modeltest 3.7 under AIC, and then BI was carried out.

Additional file 13 gives the evolutionary models, log-likelihood values (-ln L) and settings identified by Modeltest for the different datasets. In PAUP*, all six representatives of the Anseriformes were used as outgroup taxa when calculating the ML and MP trees. Only one taxon can be set as the outgroup in MrBayes. Therefore, we chose *Anseranas semipalmata*, and subsequently re-rooted the trees to make Anseriformes and Galliformes reciprocally monophyletic sister groups.

## Authors' contributions

YYS: contributed to data analysis and manuscript writing; LL: carried out the experiment work; YBS: carried out the experiment work; BSY: contributed to data analysis; XJY: contributed to data analysis; RWM: contributed to data analysis and manuscript writing; YPZ: designed the study and prepared the manuscript; All authors have read and approved the final manuscript.

## Supplementary Material

Additional file 1 **Source of sequence data**. Source of sequence data for mitochondrial genomes and general characteristics of 40 species in the Galloanserae. Click here for file

Additional file 2 **Test of stationarity of base composition**. Test of stationarity of base composition in TREEPUZZLE 5.0. The chi-square test compares the nucleotide composition of each sequence to the frequency distribution assumed in the maximum likelihood model. Click here for file

Additional file 3 **Base composition for the 12 protein-coding gene set**. (A) All codon sites; (B) 1^st ^codon position; (C) 2^nd ^codon position; (D) 3^rd ^codon position. Click here for file

Additional file 4 **Bayesian tree based on 12 mitochondrial protein-coding genes**. In order to emphasize the topology of Galliformes, we did not include the outgroup on the tree. Numbers are maximum likelihood bootstrap support and Bayesian posterior probabilities. Branches are drawn proportionally to the average number of expected DNA substitutions per site among all trees sampled after a burn-in period, as indicated by the scale at the bottom left. Species belonging to the Tribe Phasianini are marked in purple, and to Tribe Perdicini in green. Click here for file

Additional file 5 **Substitution saturation of 12 mitochondrial protein-coding genes**. Transitions and transversions plotted against the pairwise sequence divergence for 12 mitochondrial protein-coding genes. (A) 1^st ^codon position; (B) 2^nd ^codon position; (C) 3^rd ^codon position; (D) all codon sites. Click here for file

Additional file 6 **Bayesian phylogenetic analyses of RY-coding and exclusion the 3^rd ^codon position**. Bayesian phylogenetic analyses of two weighting strategies in the combined 12 protein-coding gene sets. Bayesian posterior probabilities >70% are indicated on the branches. (A) Recoding the 3^rd ^codon position nucleotides as to two-state categories, R (purine) and Y (pyrimidine), (RY-coding); (B) Excluding the 3^rd ^codon position. Click here for file

Additional file 7 **Bayesian tree for 22 tRNA genes**. Bayesian inference consensus tree for the Galliformes based on combined data from mitochondrial 22 tRNA genes. Anseriformes forms the outgroup. Bayesian posterior probabilities >70%, and maximum likelihood bootstrap proportions >50% are indicated on the branches. Click here for file

Additional file 8 **Bayesian tree based on combined data from *12S *rRNA and *16S *rRNA genes**. Bayesian inference consensus tree for Galliformes based on combined data from mitochondrial *12S *rRNA and *16S *rRNA genes. Anseriformes forms the outgroup. The Bayesian posterior probabilities >70%, and maximum likelihood bootstrap proportions >50% are indicated on the branches. Click here for file

Additional file 9 **Bayesian analyses of individual mt genes**. Bayesian inference analyses of individual mt genes and control region (CR). Each run was conducted with 5,000,000 generations and sampled every 100 generations. Bayesian Posterior Probabilities >70% are indicated on the branches. (A) *ND1*, 972 aligned sites; (B) *ND2*, 1,038 aligned sites; (C) *ND3*, 348 aligned sites; (D) *ND4L*, 291 aligned sites; (E) *ND4*, 1,377 aligned sites; (F) *ND5*, 1,818 aligned sites; (G) *ND6*, 519 aligned sites; (H) *CoxI*, 1,548 aligned sites; (I) *CoxII*, 681 aligned sites; (J) *CoxIII*, 783 aligned sites; (K) *ATP6*, 681 aligned sites; (L) *ATP8*, 165 aligned sites; (M) *CytB*, 1,137 aligned sites; (N) CR, 1,294 aligned sites; (O) *12S*, 1,047 aligned sites; (P) *16S*, 1,695 aligned sites. Click here for file

Additional file 10 **Bayesian trees based on combined datasets of the Cracidae and Odontophoridae**. Bayesian inference (BI) consensus trees based on combined datasets of the Cracidae and Odontophoridae. Anseriformes forms the outgroup. Bayesian posterior probabilities > 70% are indicated on the branches. (A) The BI tree for the dataset of 10,502 nucleotide positions for 11 species in the Cracidae (marked in red) and 40 galliform/anseriform birds that have complete mt genomes; (B) The BI tree for the dataset of 3,262 aligned nucleotide positions for 11 species in the Cracidae (marked in red), four in the Odontophoridae (marked in blue) and 40 galliform/anseriform birds. Click here for file

Additional file 11 **Source of sequence data for the Cracidae and Odontophoridae**. No complete mitochondrial genomes were available for the Cracidae and Odontophoridae in GenBank. Thus, we mined GenBank and created a dataset with as many genes as possible for them. Click here for file

Additional file 12 **Primers for amplifying the complete mitogenomes**. List of primers used in this mitogenomic study of the Galliformes. Click here for file

Additional file 13 **Parameters of evolutionary models**. Evolutionary models, log-likelihood values (-ln L), and settings identified by Modeltest for different DNA sequence datasets from the Galiformes. Click here for file
