# Supplementary material for: A mitogenomic perspective on the ancient, rapid radiation in the Galliformes with an emphasis on the Phasianidae
Source: BMC Evol Biol. 2010 May 6;10:132. doi: 10.1186/1471-2148-10-132 (PMC2880301; doi:10.1186/1471-2148-10-132)
Supplement: Additional file 1 — Source of sequence data. Source of sequence data for mitochondrial genomes and general characteristics of 40 species in the Galloanserae. [file 1471-2148-10-132-S1.DOC]

Additional file 1

Source of Sequence Data

| Taxa | | |  | Genome Length (bp) | | | | | G+C nucleotide content (%) | | | | |
| --- | --- | --- | --- | --- | --- | --- | --- | --- | --- | --- | --- | --- | --- |
| Order | Family | Scientific name | Accession  Number | Total | Protein  coding | rRNAs | tRNAs | Control  region | Total | Protein  coding | rRNAs | tRNAs | Control  region |
| Galliforms | Phasianidae | *Coturnix japonica* | NC_003408 | 16697 | 11016 | 2589 | 1546 | 1555 | 44.42 | 44.98 | 44.84 | 42.43 | 41.74 |
|  |  | *Coturnix chinensis* | NC_004575 | 16687 | 11361 | 2580 | 1550 | 1150 | 44.63 | 45.17 | 45.81 | 42.84 | 39.83 |
|  |  | *Alectoris chukar* ^a^ | FJ752426 | 16686 | 11358 | 2584 | 1539 | 1154 | 45.24 | 45.47 | 46.44 | 43.21 | 41.51 |
|  |  | *Arborophila rufogularis* ^a^ | FJ752424 | 16726 | 11362 | 2596 | 1544 | 1178 | 44.52 | 45.02 | 45.84 | 42.16 | 40.75 |
|  |  | *Arborophila gingica* ^a^ | FJ752425 | 16729 | 11366 | 2596 | 1544 | 1176 | 43.71 | 43.93 | 45.84 | 41.06 | 41.07 |
|  |  | *Bambusicola fytchii* ^a^ | FJ752423 | 16726 | 11355 | 2595 | 1542 | 1174 | 46.55 | 47.09 | 47.28 | 43.32 | 41.99 |
|  |  | *Bambusicola thoracica* ^b^ | EU165706 | 16726 | 11358 | 2596 | 1545 | 1146 | 45.56 | 46.21 | 46.73 | 42.85 | 48.34 |
|  |  | *Tetraophasis szechenyii* ^a^ | FJ752428 | 16709 | 11365 | 2583 | 1545 | 1167 | 45.92 | 46.73 | 46.46 | 42.65 | 41.30 |
|  |  | *Pucrasia macrolopha* ^a^ | FJ752429 | 16696 | 11365 | 2585 | 1542 | 1154 | 44.64 | 45.00 | 46.42 | 42.09 | 41.25 |
|  |  | *Perdix dauuricae* ^a^ | FJ752431 | 16695 | 11346 | 2571 | 1545 | 1153 | 46.04 | 46.88 | 46.87 | 43.17 | 40.68 |
|  |  | *Francolinus pintadeanus* ^b^ | EU165707 | 16690 | 11355 | 2579 | 1544 | 1169 | 45.20 | 45.78 | 46.10 | 43.65 | 40.29 |
|  |  | *Tragopan temminckii* ^a^ | FJ752427 | 16728 | 11352 | 2592 | 1556 | 1178 | 45.34 | 46.01 | 46.14 | 43.06 | 41.09 |
|  |  | *Lophophorus sclateri* ^a^ | FJ752432 | 16707 | 11349 | 2581 | 1542 | 1167 | 44.24 | 44.65 | 45.06 | 42.74 | 40.70 |
|  |  | *Gallus gallus* | [NC_007236](http://www.ncbi.nlm.nih.gov/entrez/viewer.fcgi?db=nucleotide&val=71658078) | 16785 | 11358 | 2598 | 1546 | 1232 | 46.01 | 47.09 | 46.54 | 43.14 | 40.10 |
|  |  | *Gallus sonneratii* | NC_007240 | 16841 | 11358 | 2491 | 1534 | 1292 | 45.96 | 46.80 | 47.41 | 43.22 | 40.17 |
|  |  | *Gallus lafayettei* | NC_007239 | 16841 | 11358 | 2597 | 1539 | 1292 | 45.86 | 46.82 | 46.67 | 42.76 | 39.86 |
|  |  | *Gallus varius* | NC_007238 | 16783 | 11358 | 2595 | 1545 | 1228 | 45.92 | 46.88 | 46.63 | 42.72 | 40.07 |
|  |  | *Lophura nycthemera* ^b^ | EU417810 | 16680 | 11358 | 2576 | 1546 | 1148 | 45.24 | 46.08 | 45.69 | 41.40 | 41.03 |
|  |  | *Lophura ignita* | AB164627 | 16688 | 11358 | 2575 | 1552 | 1147 | 44.94 | 45.62 | 45.44 | 41.82 | 41.50 |
|  |  | *Phasianus colchicus* ^a^ | FJ752430 | 16683 | 11365 | 2583 | 1547 | 1144 | 44.23 | 44.88 | 45.26 | 41.31 | 40.21 |
|  |  | *Phasianus versicolor* | AB164626 | 16690 | 11358 | 2586 | 1547 | 1150 | 44.04 | 44.59 | 45.24 | 41.24 | 40.43 |
|  |  | *Chrysolophus pictus* ^a^ | FJ752433 | 16678 | 11358 | 2574 | 1542 | 1152 | 44.80 | 45.24 | 45.92 | 42.22 | 40.71 |
|  |  | *Chrysolophus amherstiae* ^a^ | FJ752434 | 16678 | 11366 | 2574 | 1542 | 1148 | 44.72 | 45.15 | 45.92 | 42.61 | 41.11 |
|  |  | *Polyplectron bicalcaratum* ^b^ | EU417812 | 16699 | 11358 | 2576 | 1546 | 1170 | 46.57 | 47.31 | 47.83 | 43.73 | 40.94 |
|  |  | *Pavo muticus* ^b^ | EU417811 | 16698 | 11358 | 2588 | 1540 | 1156 | 44.65 | 45.01 | 46.14 | 42.34 | 41.61 |
|  |  | *Syrmaticus humiae* | AB164625 | 16686 | 11358 | 2583 | 1536 | 1153 | 44.34 | 44.87 | 45.45 | 41.80 | 40.59 |
|  |  | *Syrmaticus ellioti* | AB164624 | 16688 | 11358 | 2577 | 1553 | 1153 | 44.36 | 44.85 | 45.56 | 41.79 | 40.76 |
|  |  | *Syrmaticus reevesii* | AB164623 | 16678 | 11358 | 2574 | 1553 | 1150 | 45.35 | 45.83 | 46.08 | 42.88 | 42.78 |
|  |  | *Syrmaticus soemmerringi* | AB164622 | 16690 | 11358 | 2579 | 1549 | 1152 | 44.19 | 44.74 | 44.82 | 42.16 | 40.89 |
|  | Tetraonidae | *Bonasa bonasia* ^a^ | FJ752435 | 16673 | 11349 | 2578 | 1546 | 1141 | 44.09 | 44.61 | 44.69 | 42.50 | 40.58 |
|  | Meleagrididae | *Meleagris gallopavo* | NC_010195 | 16717 | 11361 | 2591 | 1541 | 1164 | 43.52 | 44.10 | 43.88 | 42.05 | 40.21 |
|  | Numididae | *Numida meleagris* | [NC_006382](http://www.ncbi.nlm.nih.gov/entrez/viewer.fcgi?db=nucleotide&val=54310668) | 16726 | 11343 | 2604 | 1551 | 1169 | 46.27 | 47.27 | 47.20 | 41.91 | 40.80 |
|  |  | *Acryllium vulturinum* ^a^ | FJ752436 | 16746 | 11340 | 2614 | 1558 | 1169 | 45.82 | 46.69 | 46.60 | 41.72 | 41.32 |
|  | Megapodiidae | *Alectura lathami* | [NC_007227](http://www.ncbi.nlm.nih.gov/entrez/viewer.fcgi?db=nucleotide&val=71658022) | 16698 | 11361 | 2595 | 1559 | 1120 | 47.32 | 48.04 | 47.94 | 43.23 | 44.64 |
| Anseriformes | Anatidae | *Cygnus columbianus* | NC_007691 | 16728 | 11367 | 2598 | 1535 | 1159 | 47.12 | 47.81 | 46.77 | 43.13 | 46.33 |
|  |  | *Anser albifrons* | [NC_004539](http://www.ncbi.nlm.nih.gov/entrez/viewer.fcgi?db=nucleotide&val=27819328) | 16737 | 11367 | 2597 | 1541 | 1174 | 47.22 | 48.24 | 46.48 | 42.44 | 45.40 |
|  |  | *Branta canadensis* | NC_007011 | 16760 | 11373 | 2591 | 1540 | 1194 | 47.21 | 48.28 | 46.70 | 42.34 | 44.97 |
|  |  | *Aythya americana* | NC_000877 | 16616 | 11373 | 2586 | 1544 | 1066 | 48.38 | 49.48 | 46.79 | 43.91 | 47.47 |
|  |  | *Anas platyrhynchos* | NC_009684 | 16604 | 11373 | 2587 | 1549 | 1049 | 48.59 | 49.85 | 46.93 | 43.51 | 46.81 |
|  | Anseranatidae | *Anseranas semipalmata* | NC_005933 | 16870 | 11364 | 2567 | 1547 | 1335 | 45.58 | 45.93 | 47.14 | 42.34 | 43.75 |

^a^ mitochondrial genomes from this study.

^b^ mitochondrial genomes from our another study (1).

1. Shen YY, Shi P, Sun YB, Zhang YP (2009) Relaxation of selective constraint on avian mitochondrial DNA following the degeneration of flight ability*. Genome R*es 19:1760-1765.
