# Supplementary figures and images for: A mitogenomic perspective on the ancient, rapid radiation in the Galliformes with an emphasis on the Phasianidae
Source: BMC Evol Biol. 2010 May 6;10:132. doi: 10.1186/1471-2148-10-132 (PMC2880301; doi:10.1186/1471-2148-10-132)

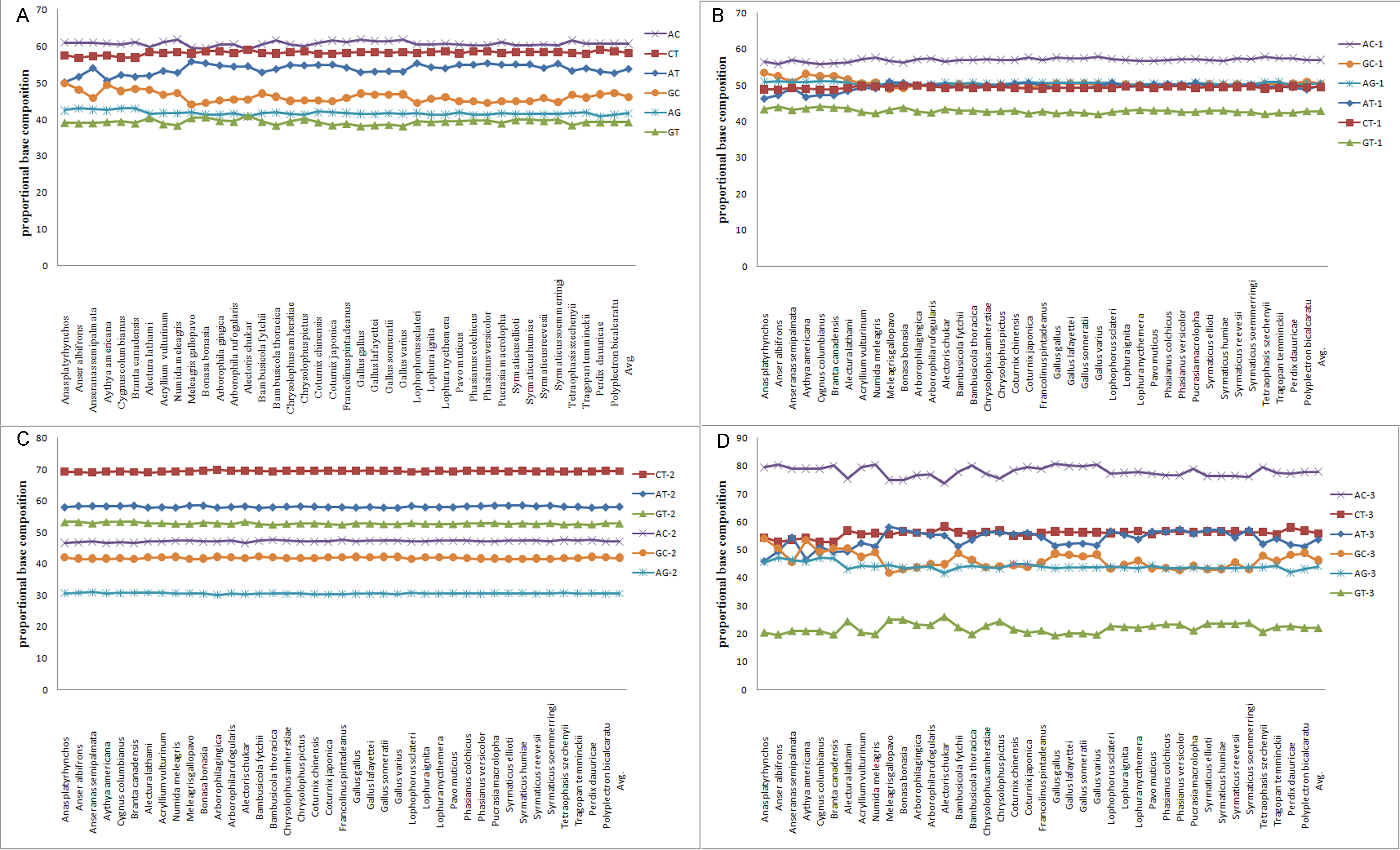

Supplement: Additional file 3 — Base composition for the 12 protein-coding gene set. (A) All codon sites; (B) 1st codon position; (C) 2nd codon position; (D) 3rd codon position. [file 1471-2148-10-132-S3.TIFF]

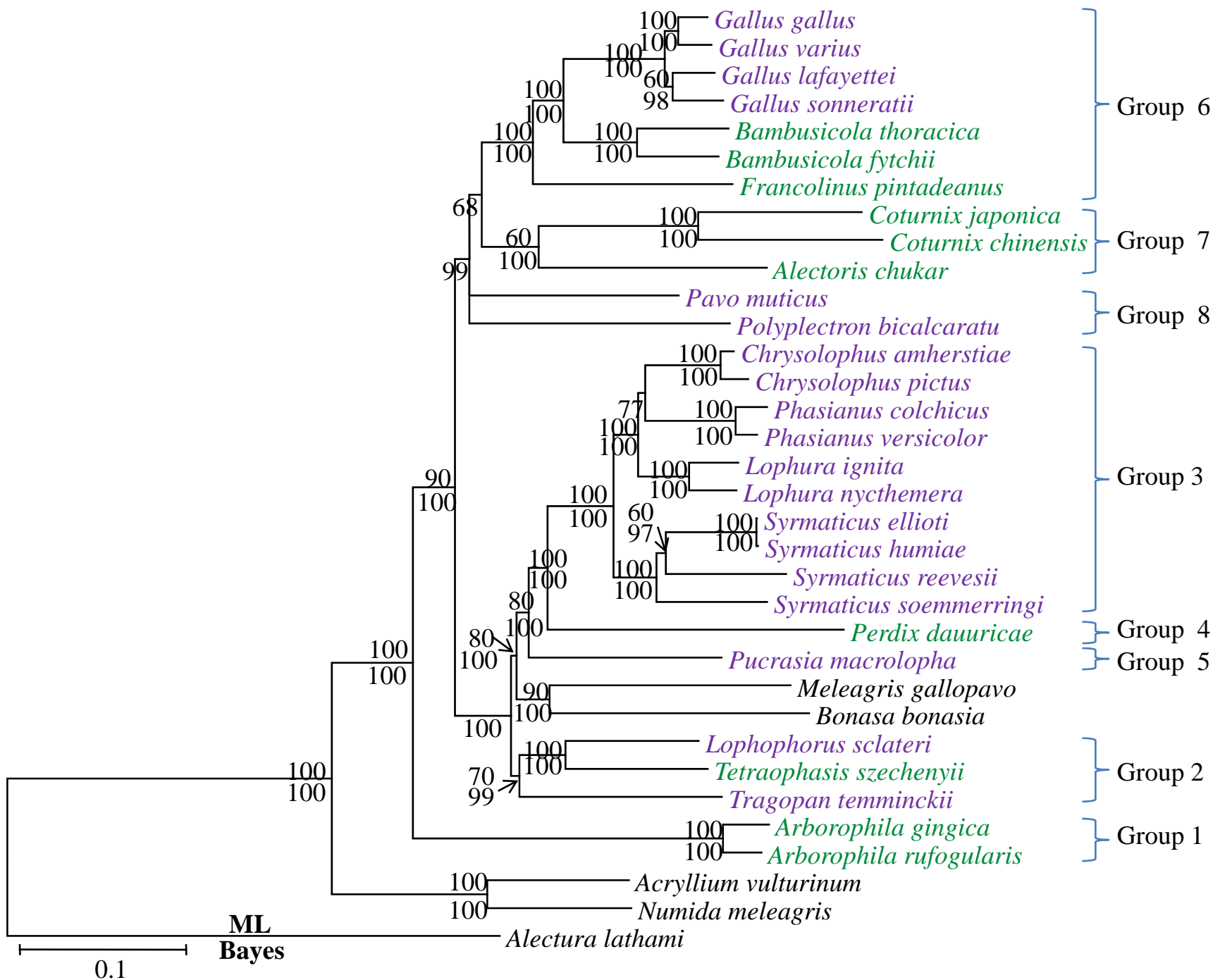

Supplement: Additional file 4 — Bayesian tree based on 12 mitochondrial protein-coding genes. In order to emphasize the topology of Galliformes, we did not include the outgroup on the tree. Numbers are maximum likelihood bootstrap support and Bayesian posterior probabilities. Branches are drawn proportionally to the average number of expected DNA substitutions per site among all trees sampled after a burn-in period, as indicated by the scale at the bottom left. Species belonging to the Tribe Phasianini are marked in purple, and to Tribe Perdicini in green. [file 1471-2148-10-132-S4.PDF]

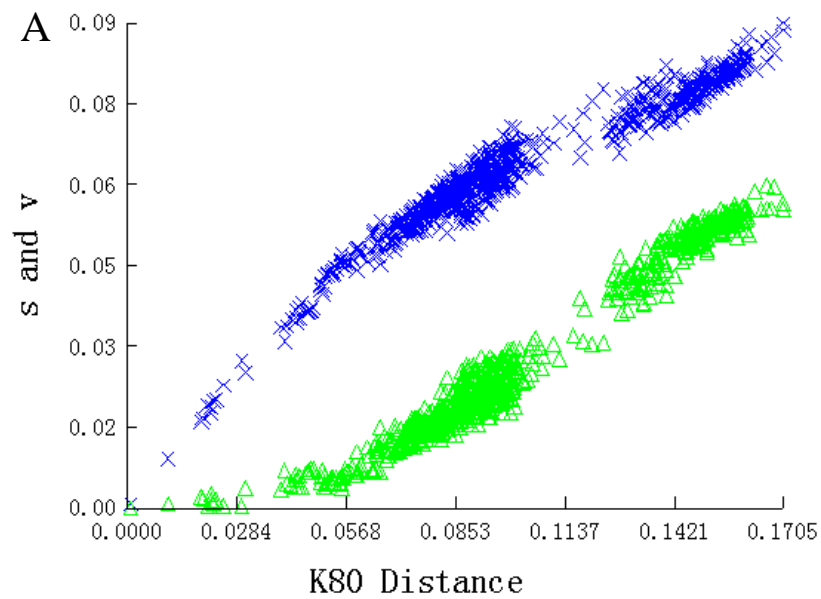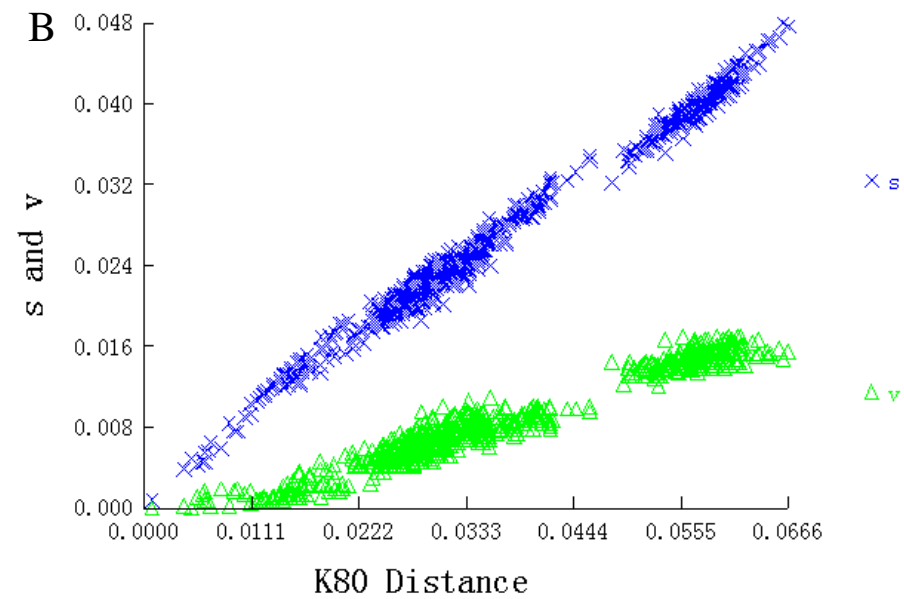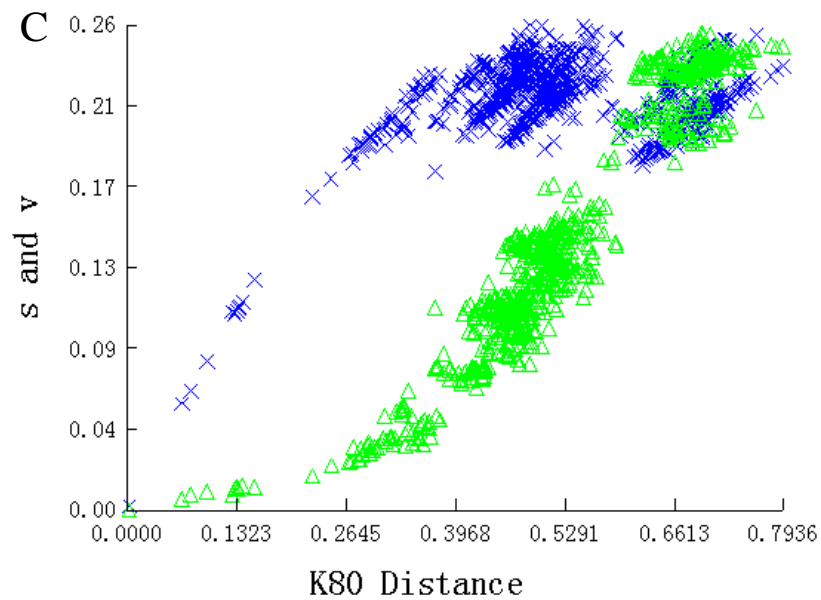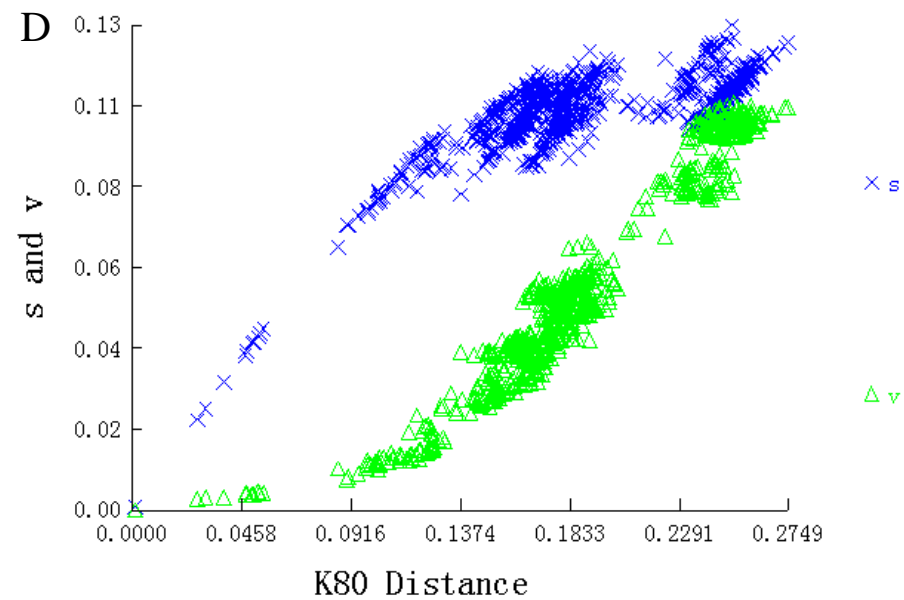

Substitution saturation

Supplement: Additional file 5 — Substitution saturation of 12 mitochondrial protein-coding genes. Transitions and transversions plotted against the pairwise sequence divergence for 12 mitochondrial protein-coding genes. (A) 1st codon position; (B) 2nd codon position; (C) 3rd codon position; (D) all codon sites. [file 1471-2148-10-132-S5.PDF]

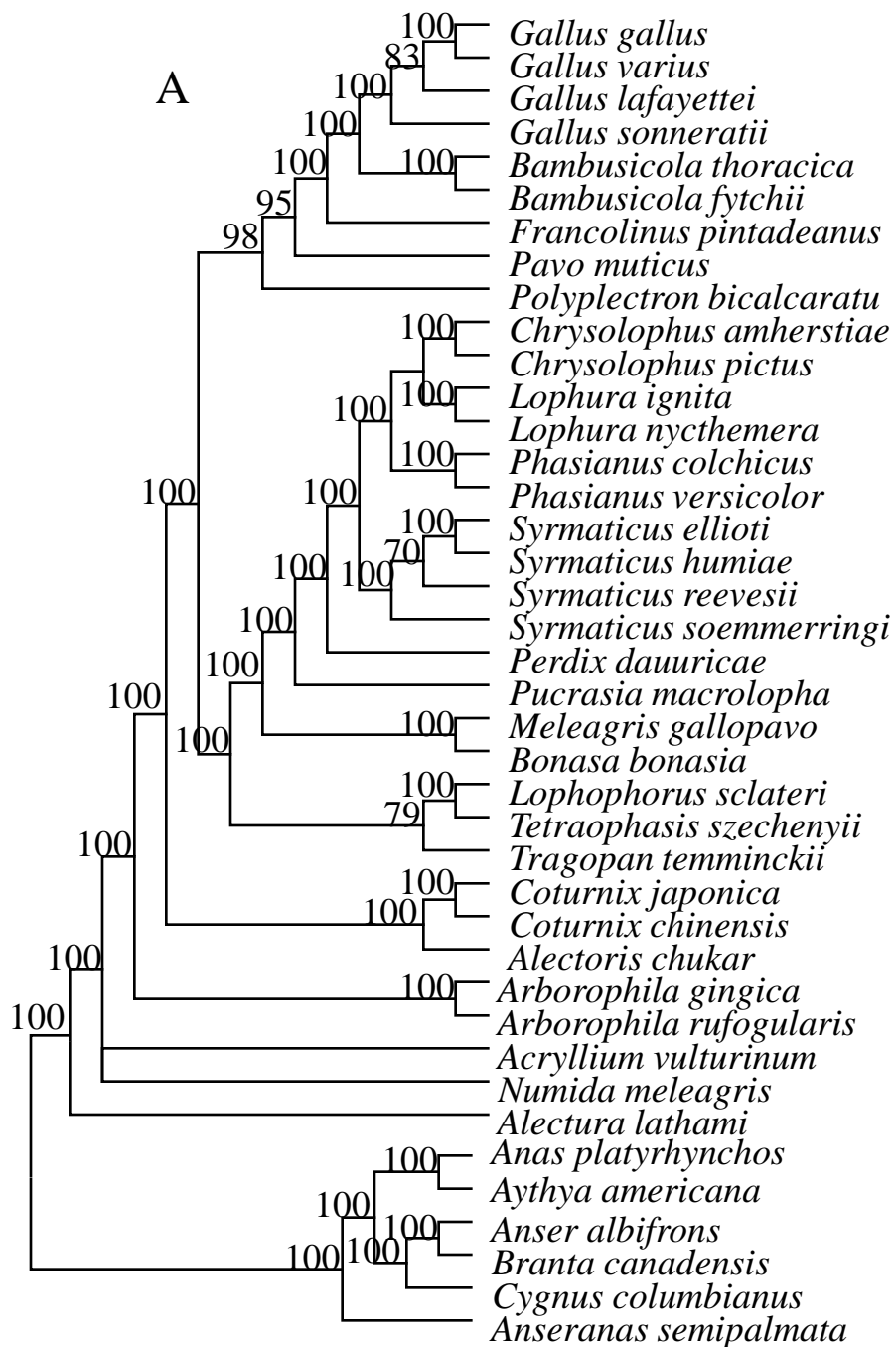

12 genes RY coding

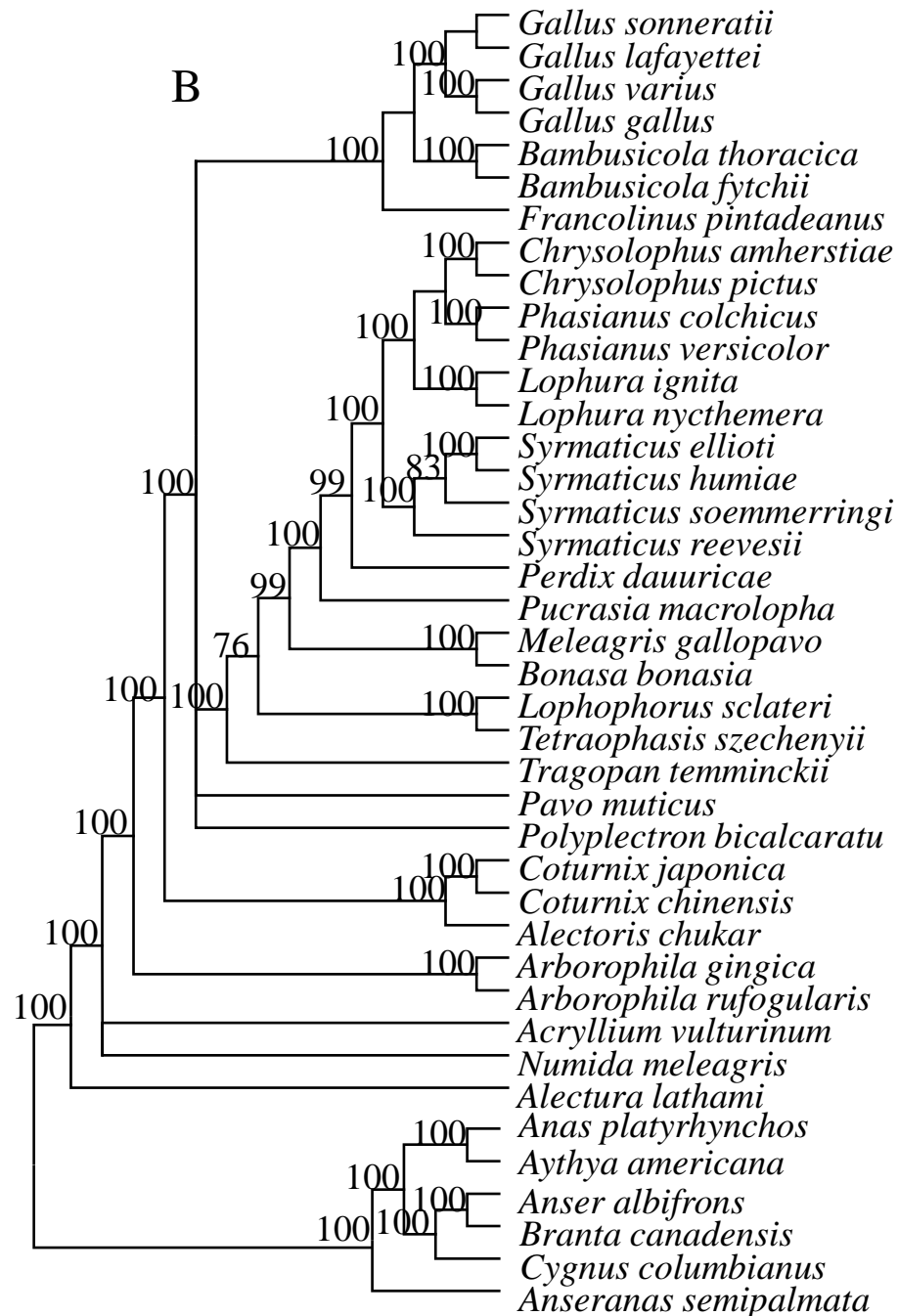

12 genes excluding 3<sup>rd</sup> codon

Supplement: Additional file 6 — Bayesian phylogenetic analyses of RY-coding and exclusion the 3rd codon position. Bayesian phylogenetic analyses of two weighting strategies in the combined 12 protein-coding gene sets. Bayesian posterior probabilities >70% are indicated on the branches. (A) Recoding the 3rd codon position nucleotides as to two-state categories, R (purine) and Y (pyrimidine), (RY-coding); (B) Excluding the 3rd codon position. [file 1471-2148-10-132-S6.PDF]

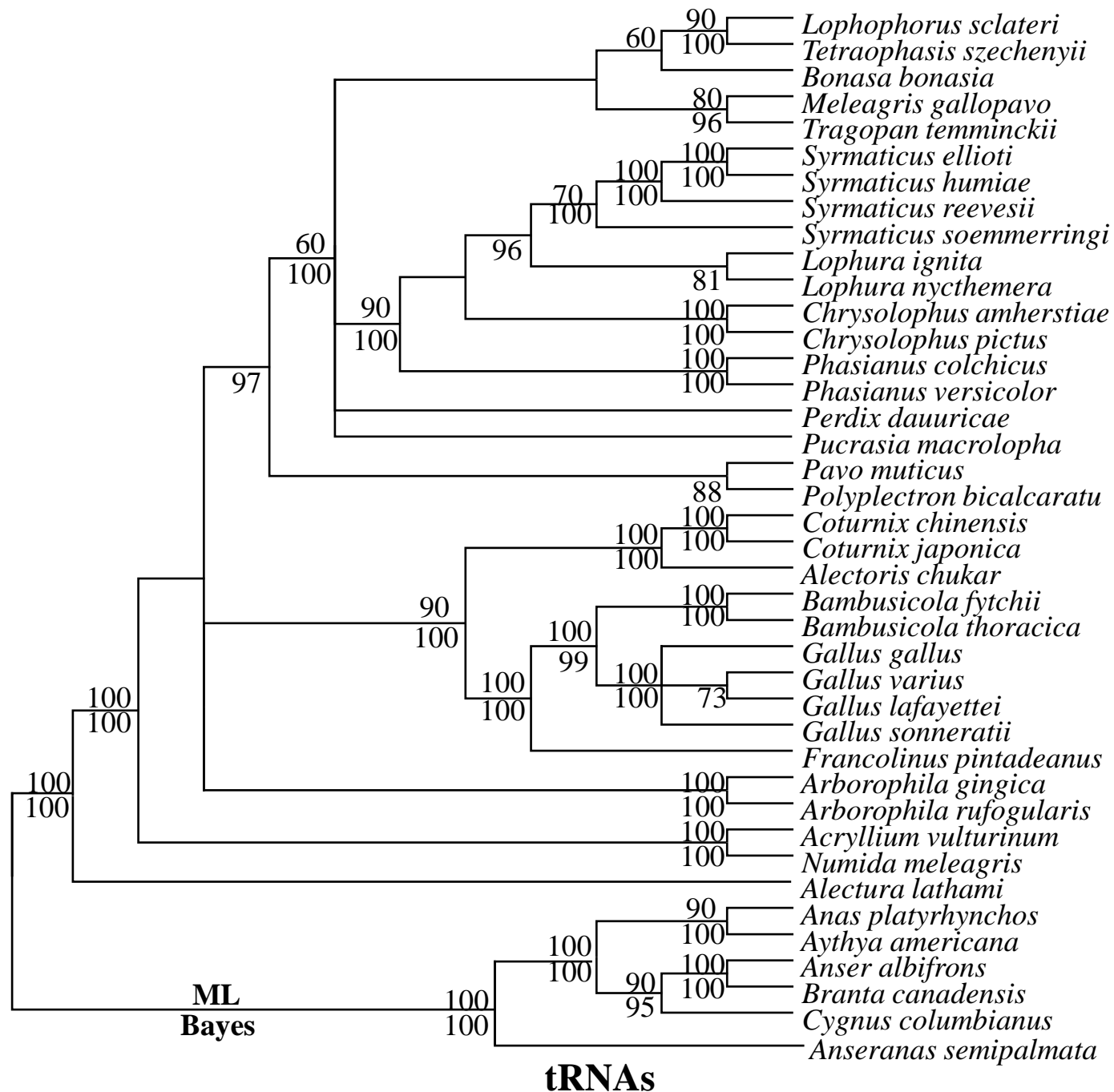

Supplement: Additional file 7 — Bayesian tree for 22 tRNA genes. Bayesian inference consensus tree for the Galliformes based on combined data from mitochondrial 22 tRNA genes. Anseriformes forms the outgroup. Bayesian posterior probabilities >70%, and maximum likelihood bootstrap proportions >50% are indicated on the branches. [file 1471-2148-10-132-S7.PDF]

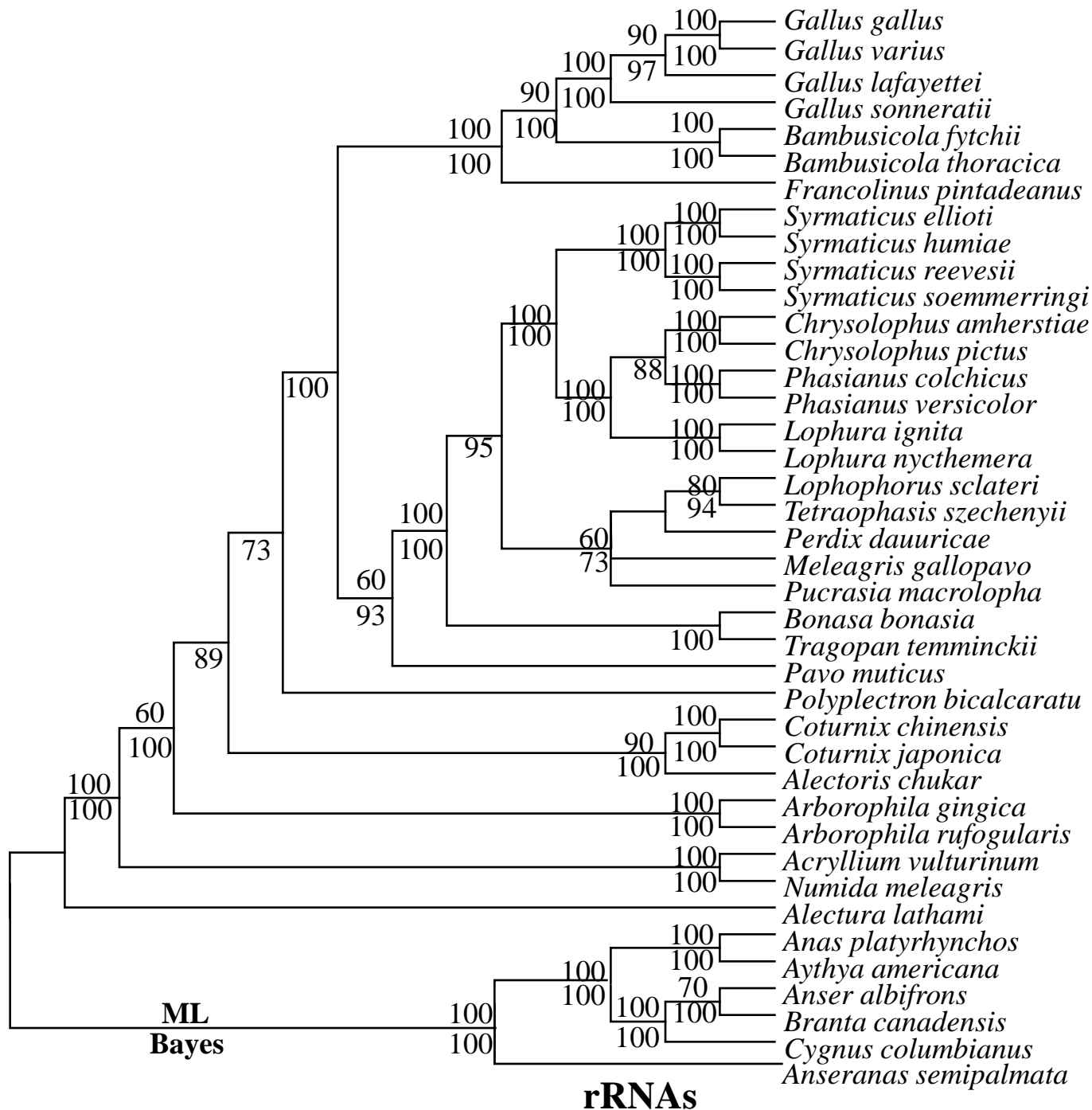

Supplement: Additional file 8 — Bayesian tree based on combined data from 12S rRNA and 16S rRNA genes. Bayesian inference consensus tree for Galliformes based on combined data from mitochondrial 12S rRNA and 16S rRNA genes. Anseriformes forms the outgroup. The Bayesian posterior probabilities >70%, and maximum likelihood bootstrap proportions >50% are indicated on the branches. [file 1471-2148-10-132-S8.PDF]

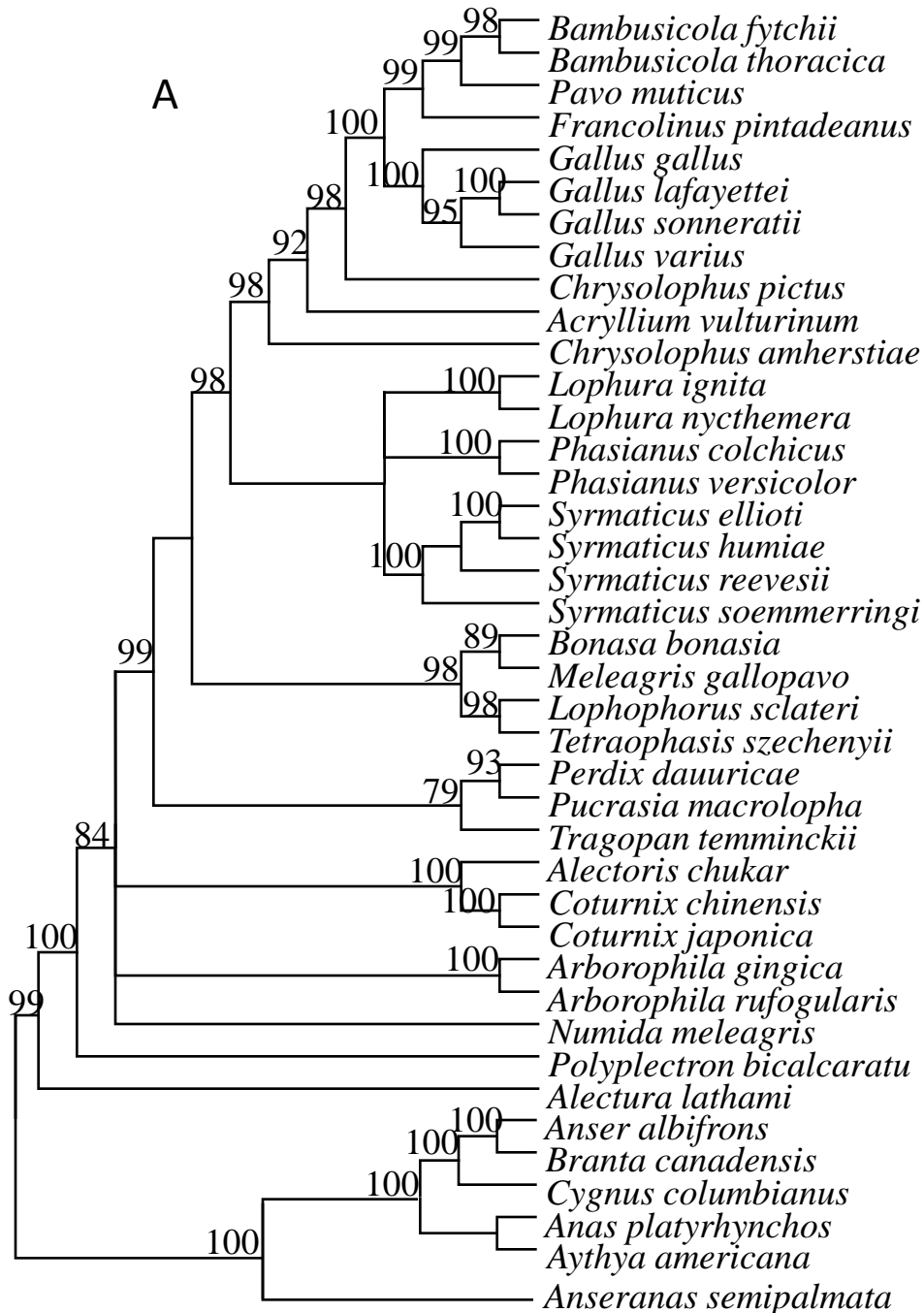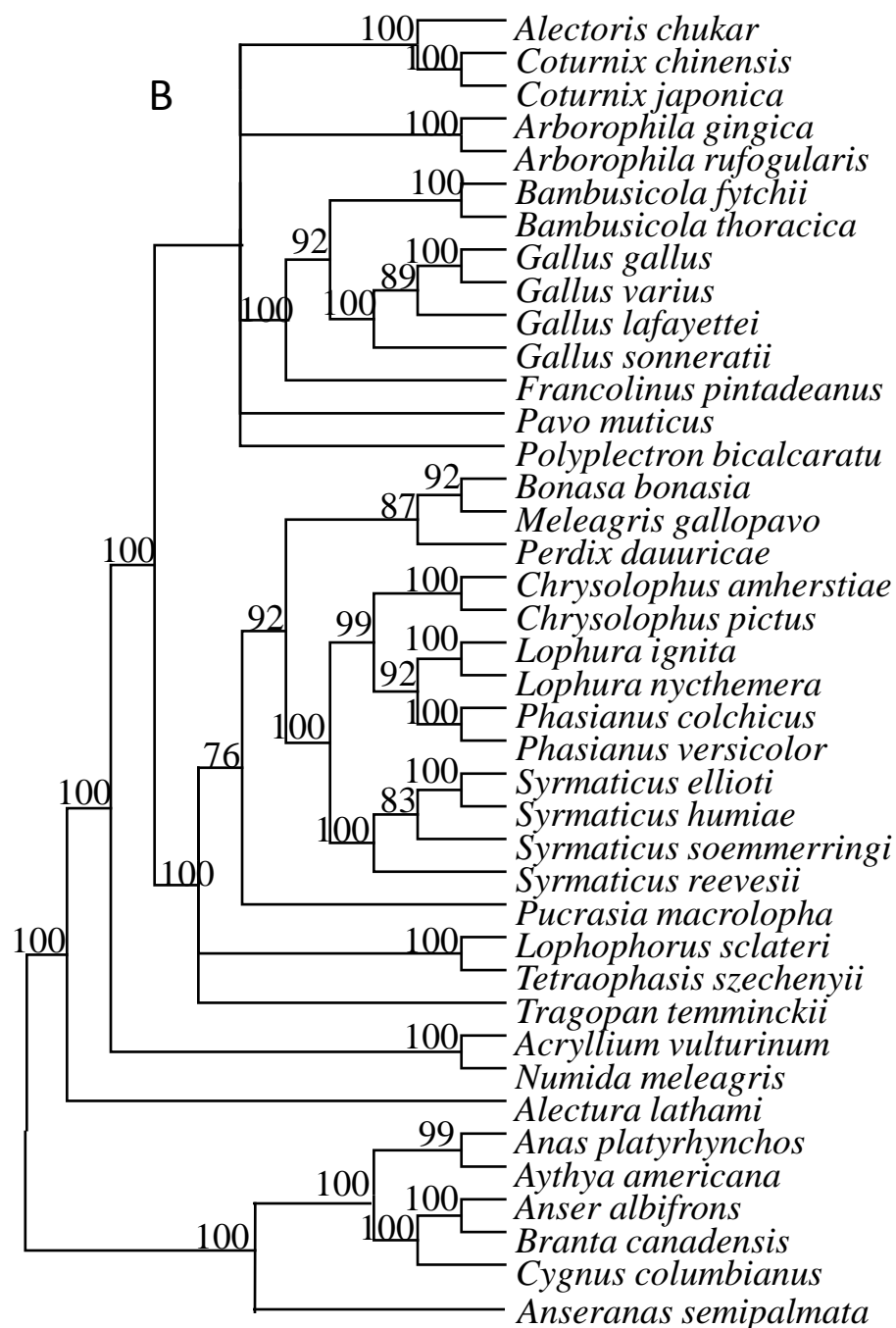

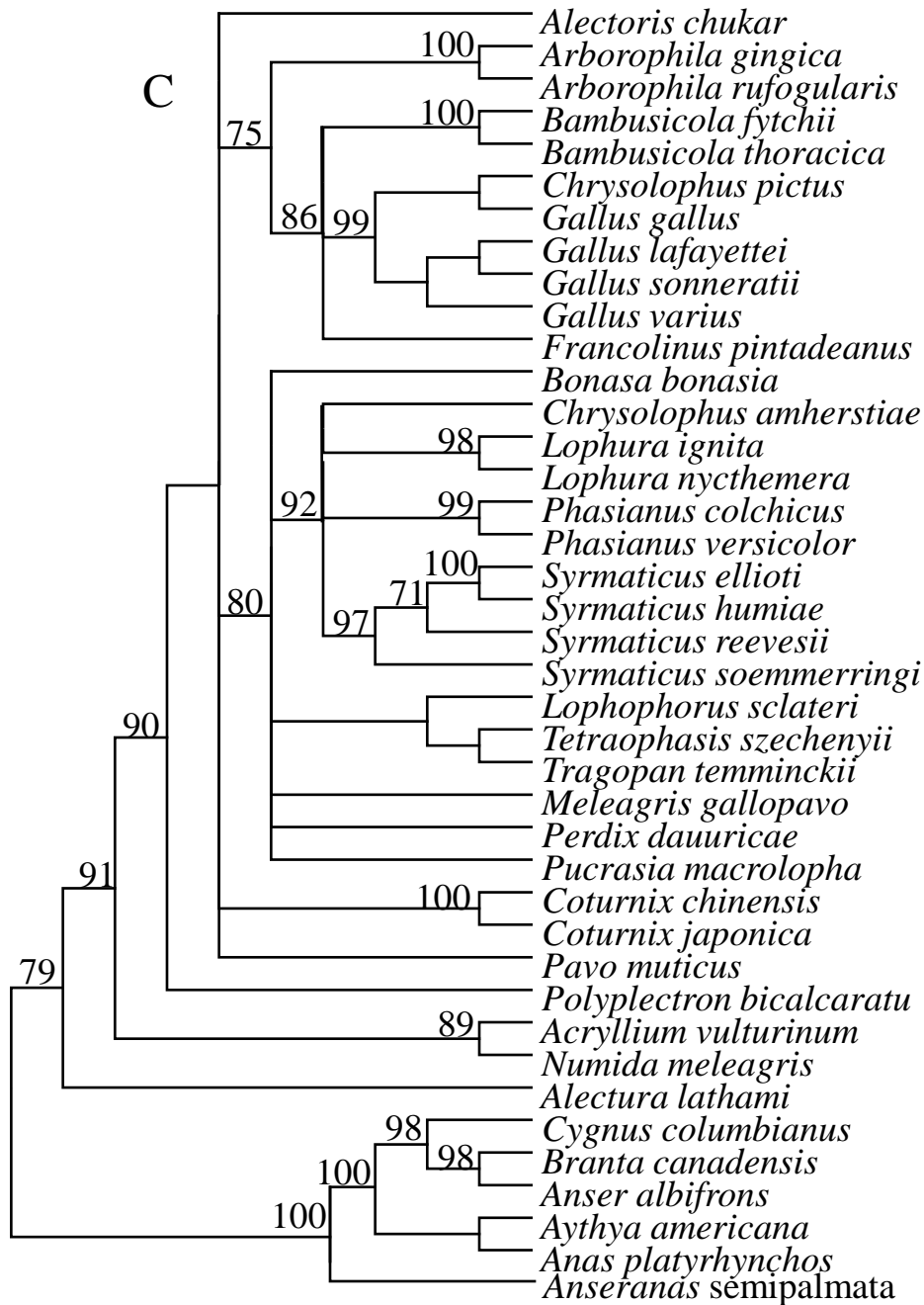

ND3

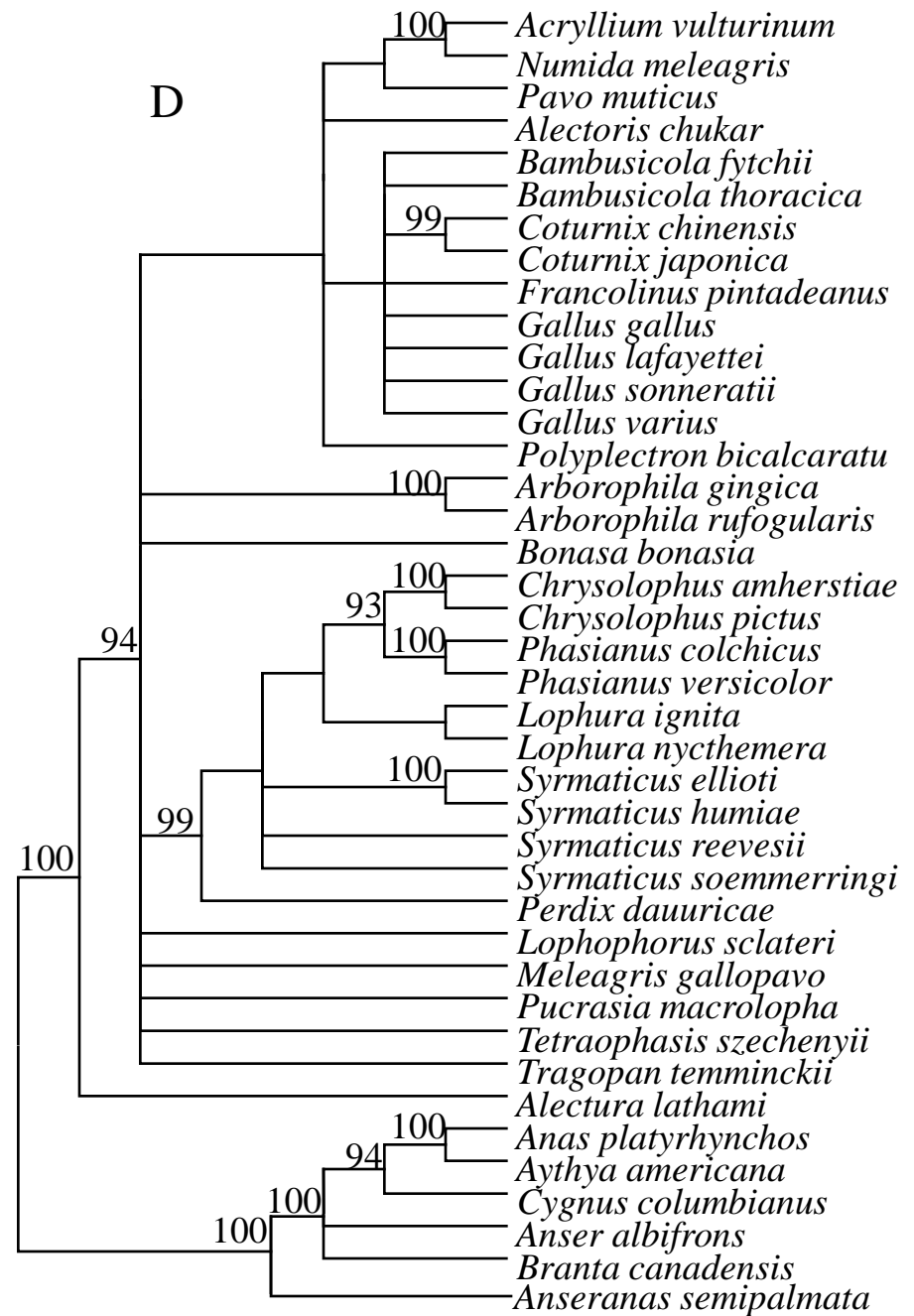

ND4L

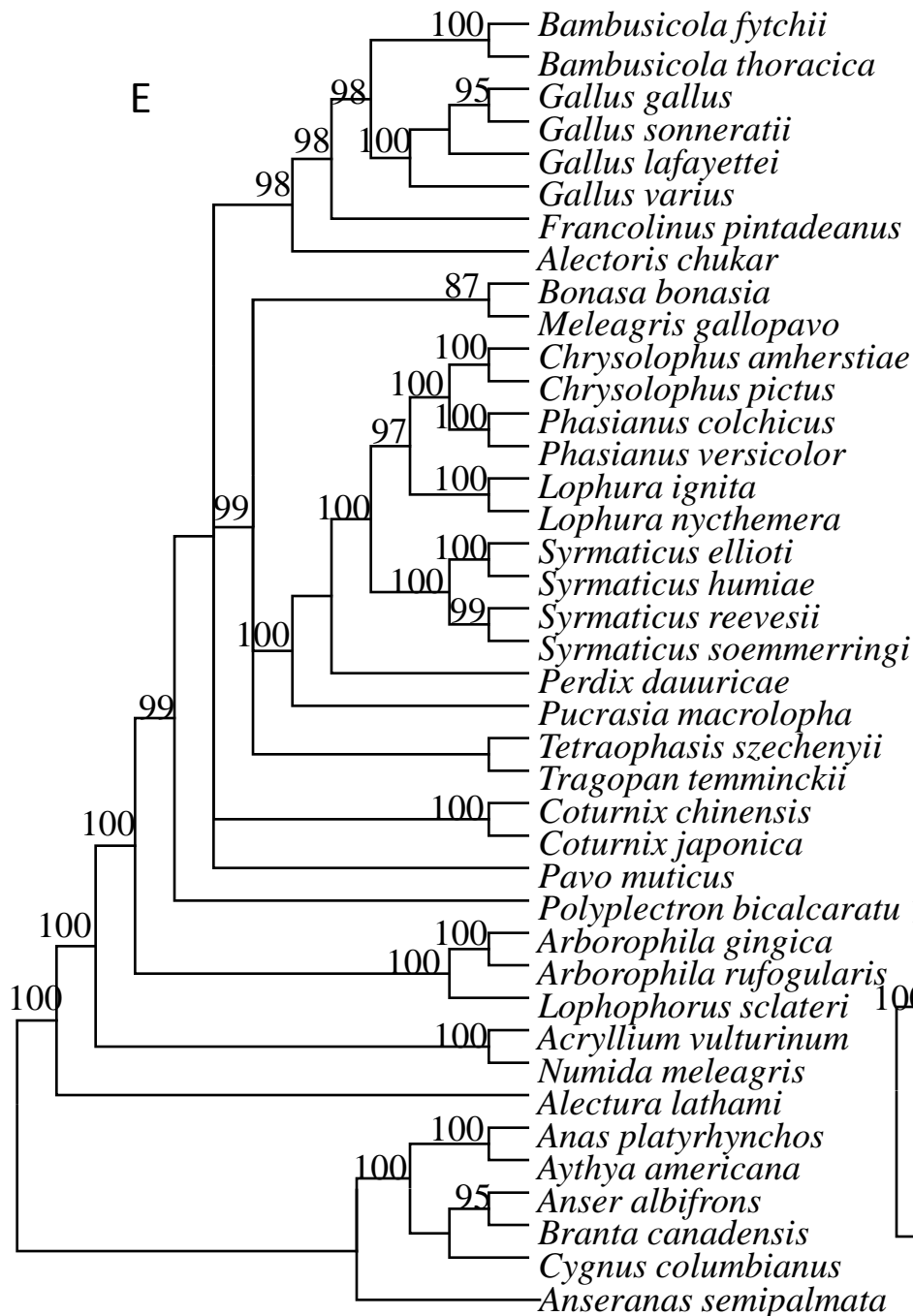

ND4

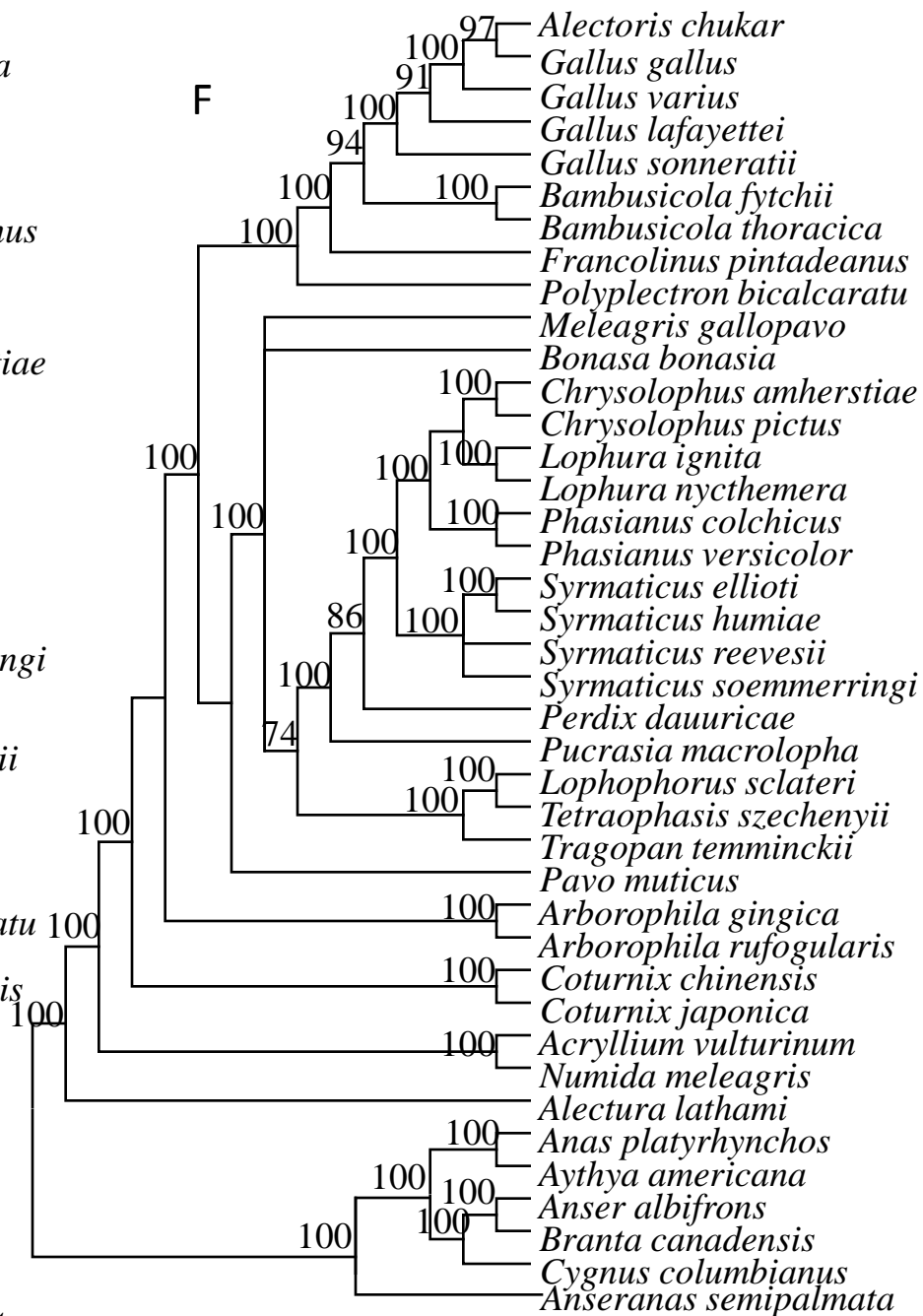

ND5

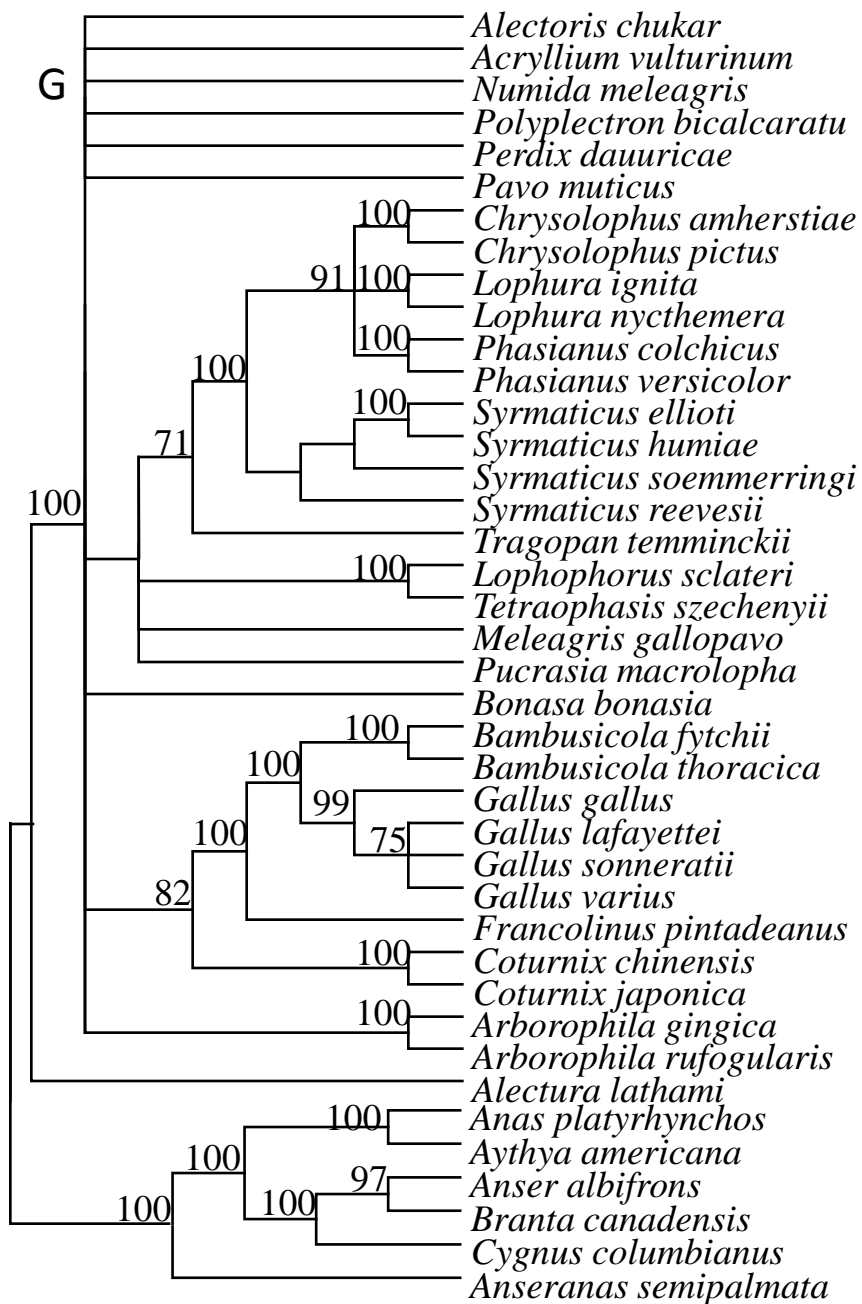

ND6

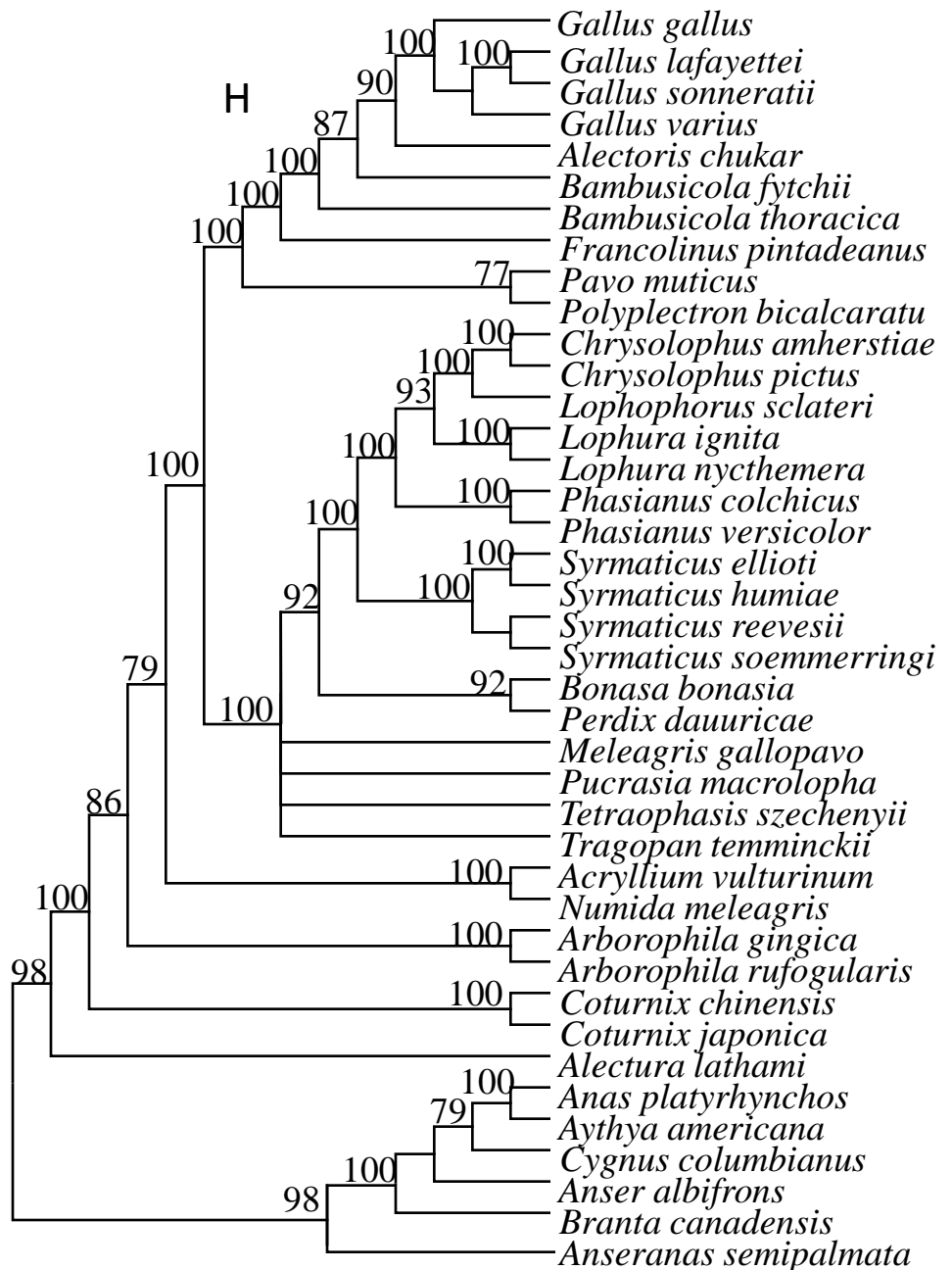

CoxI

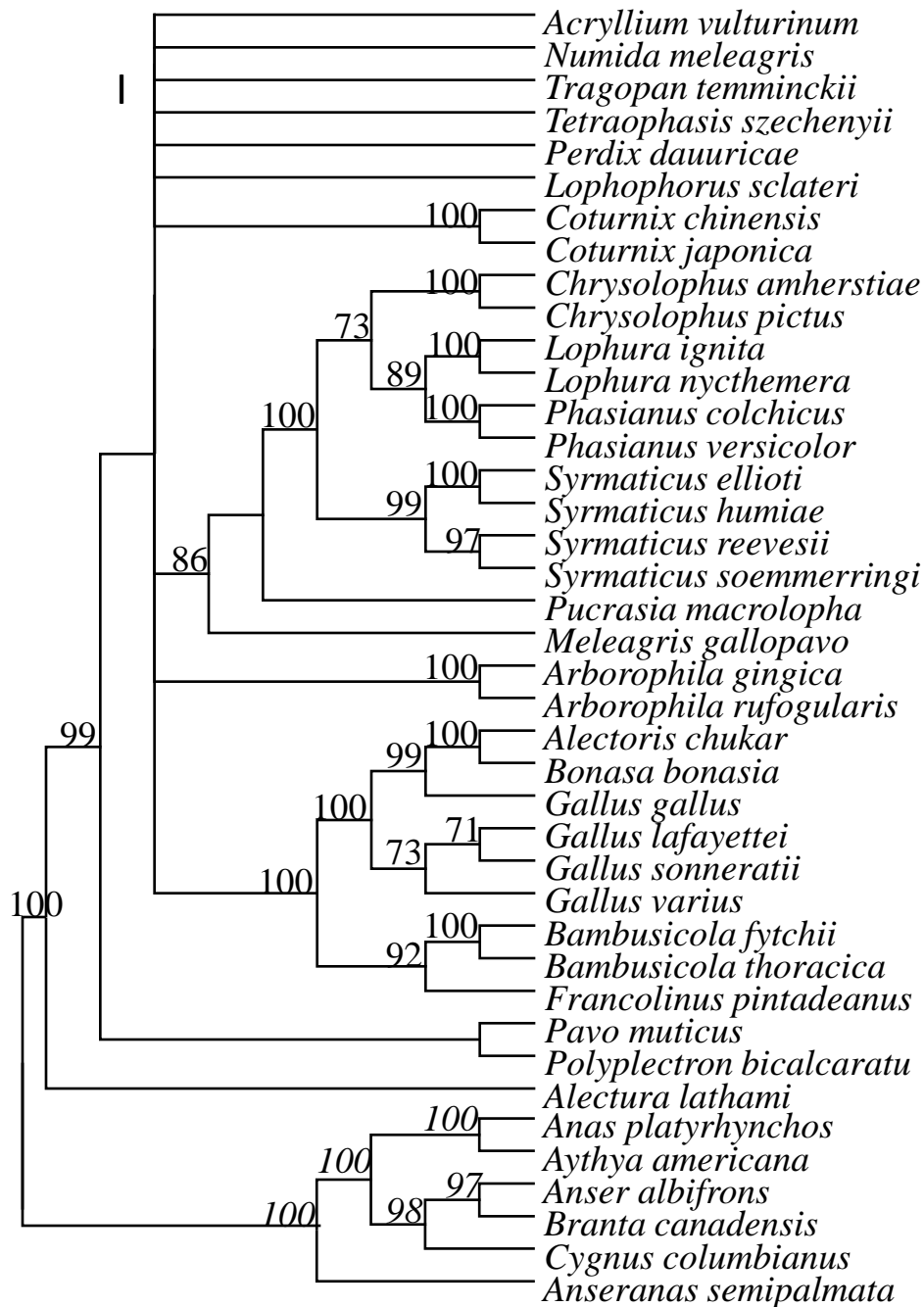

CoxII

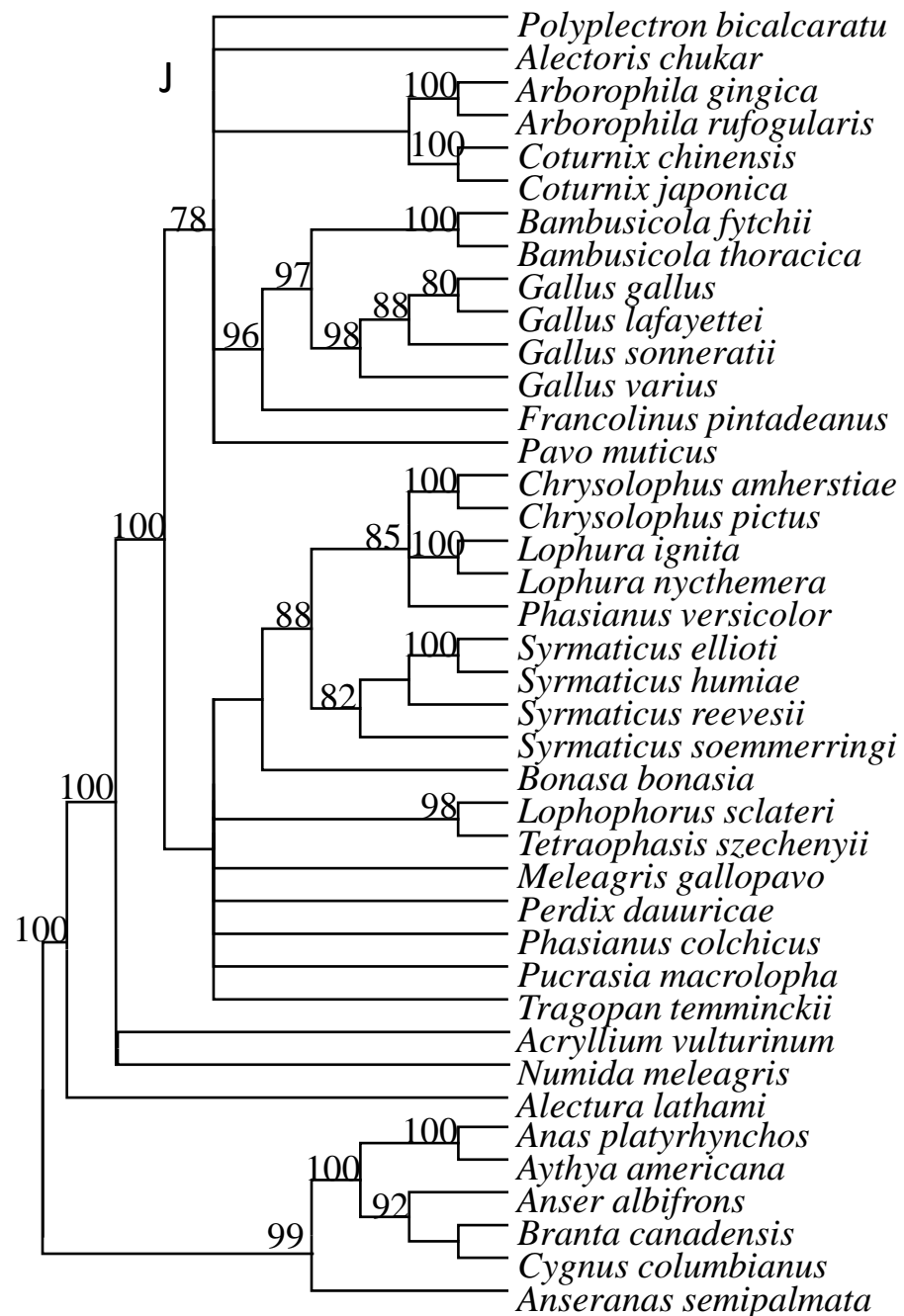

CoxIII

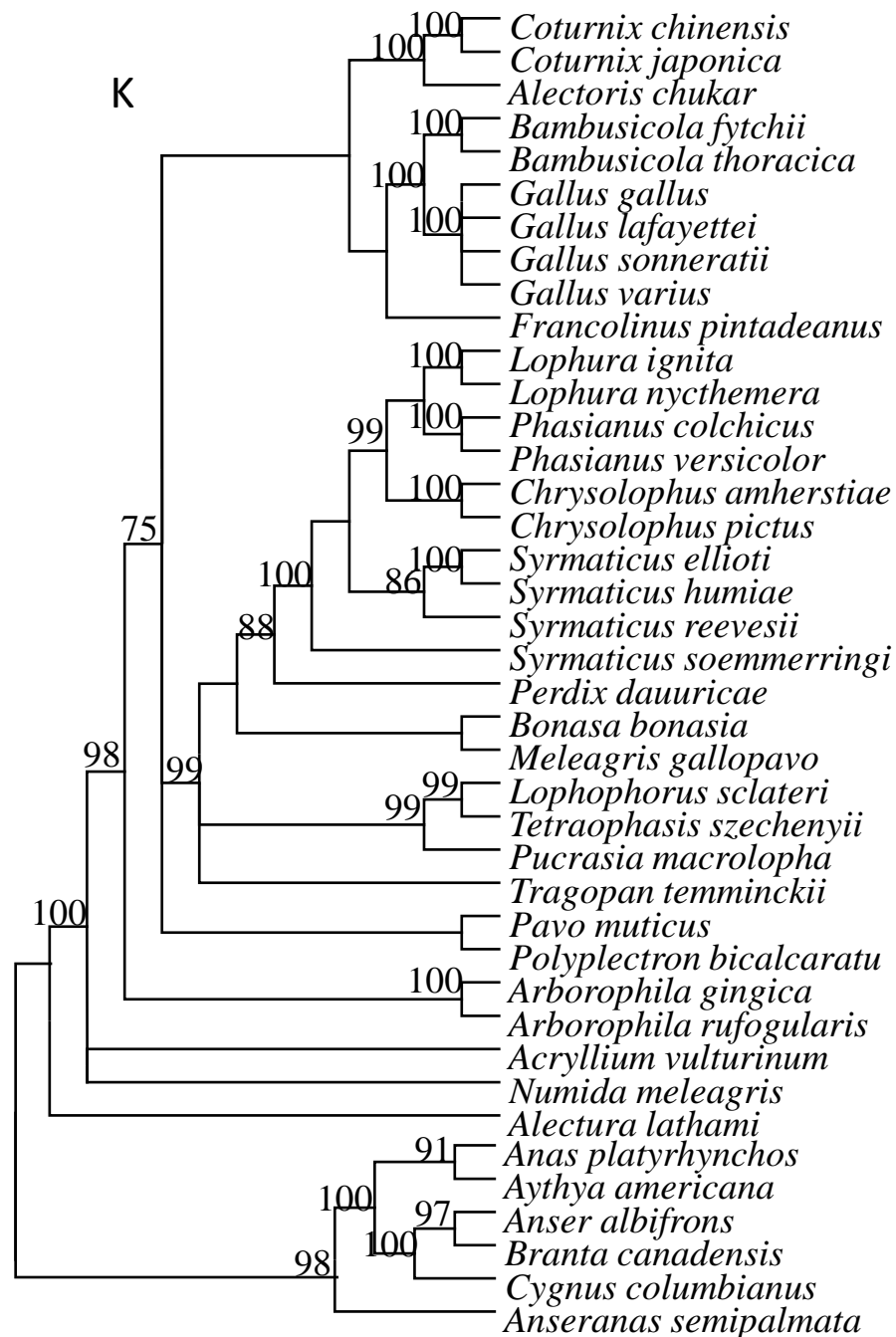

ATP6

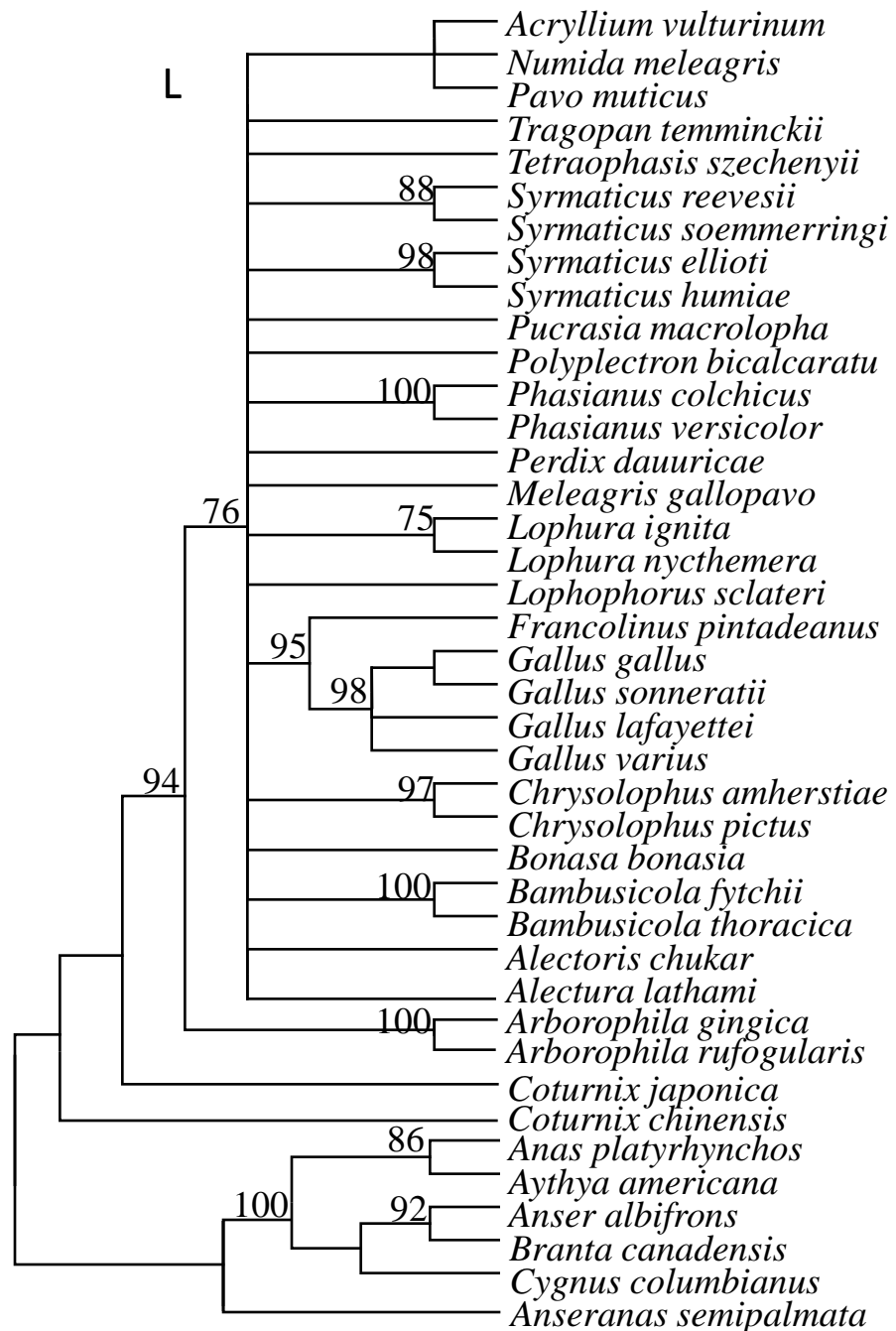

ATP8

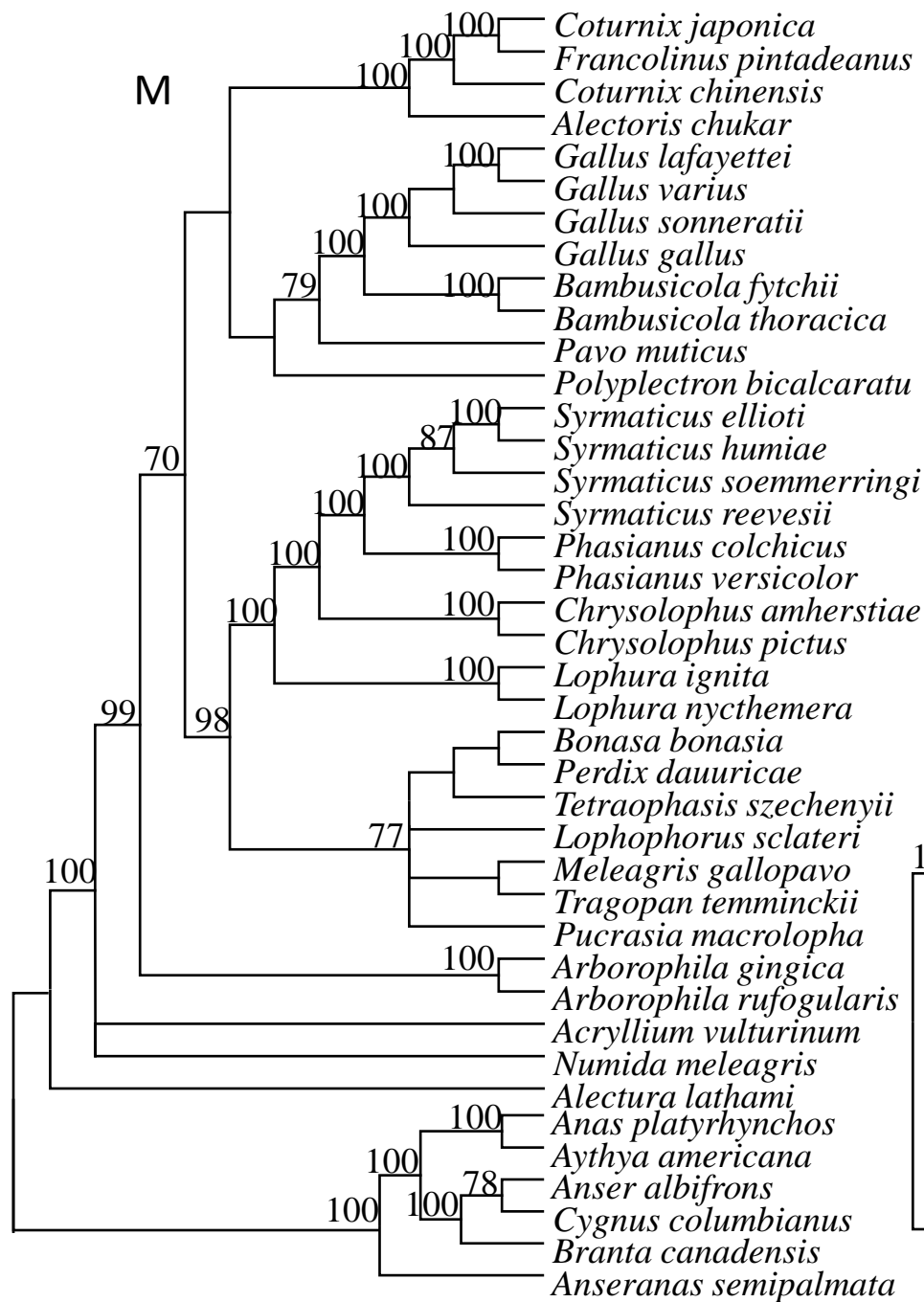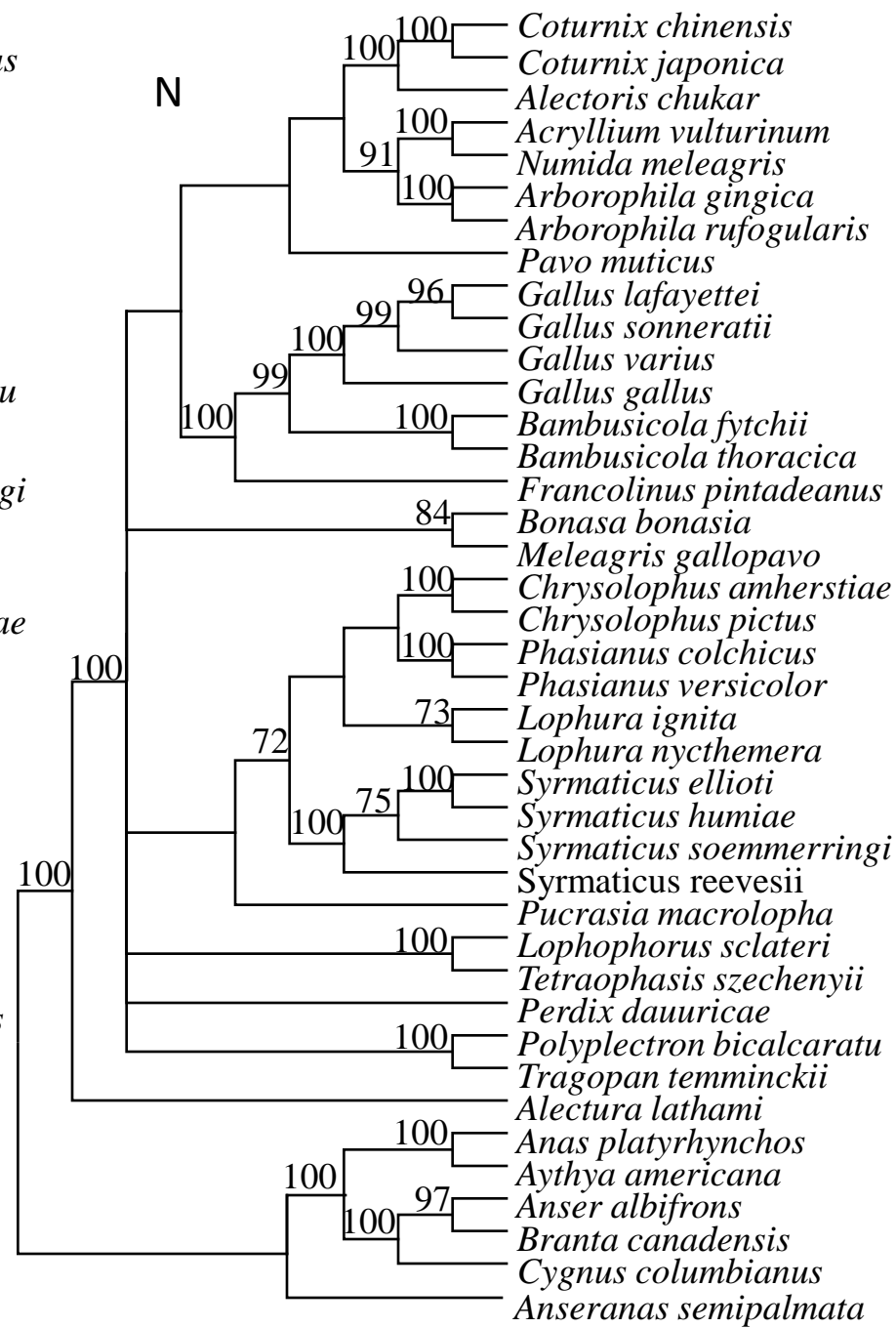

O

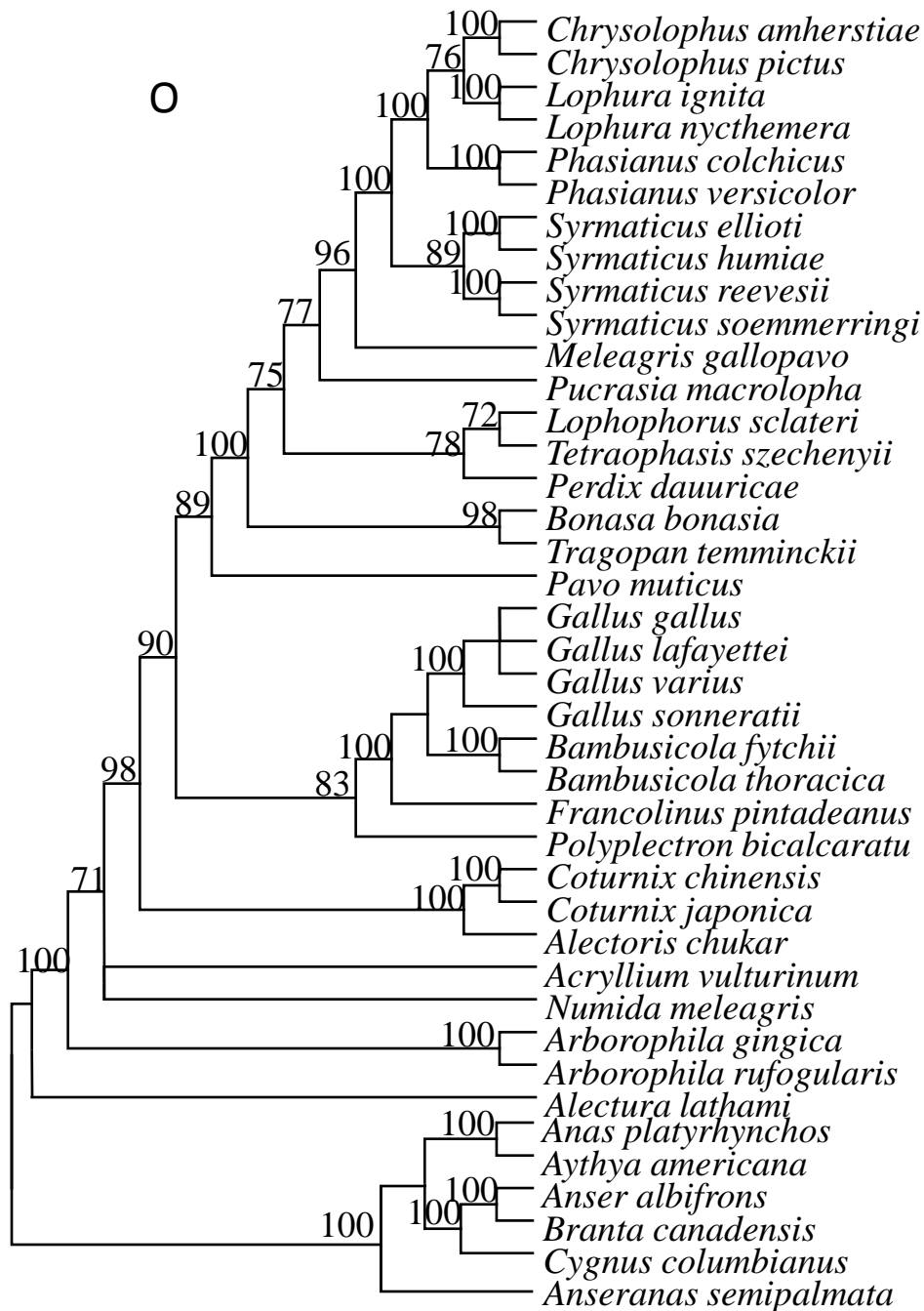

12S

P

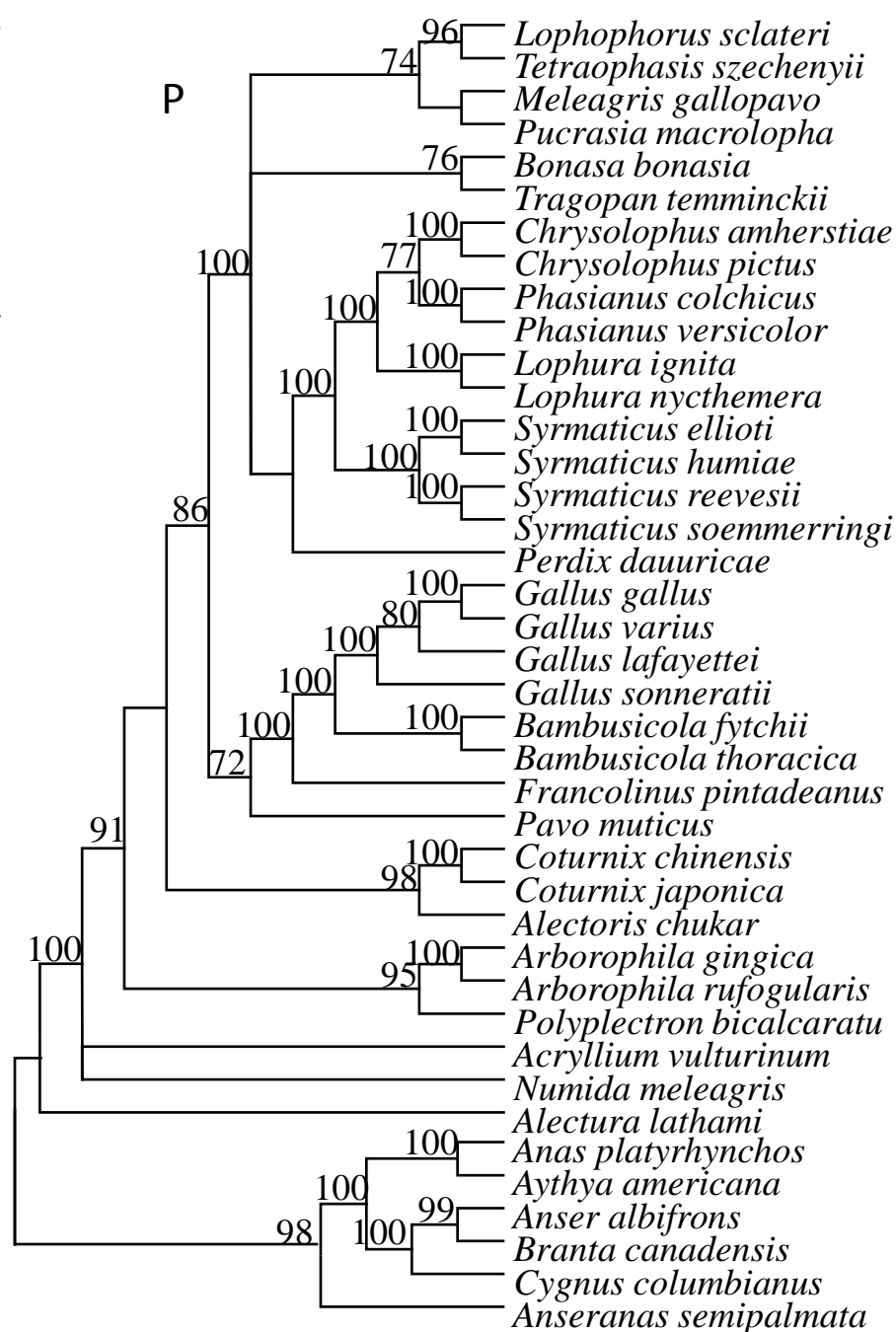

16S

Supplement: Additional file 9 — Bayesian analyses of individual mt genes. Bayesian inference analyses of individual mt genes and control region (CR). Each run was conducted with 5,000,000 generations and sampled every 100 generations. Bayesian Posterior Probabilities >70% are indicated on the branches. (A) ND1, 972 aligned sites; (B) ND2, 1,038 aligned sites; (C) ND3, 348 aligned sites; (D) ND4L, 291 aligned sites; (E) ND4, 1,377 aligned sites; (F) ND5, 1,818 aligned sites; (G) ND6, 519 aligned sites; (H) CoxI, 1,548 aligned sites; (I) CoxII, 681 aligned sites; (J) CoxIII, 783 aligned sites; (K) ATP6, 681 aligned sites; (L) ATP8, 165 aligned sites; (M) CytB, 1,137 aligned sites; (N) CR, 1,294 aligned sites; (O) 12S, 1,047 aligned sites; (P) 16S, 1,695 aligned sites. [file 1471-2148-10-132-S9.PDF]

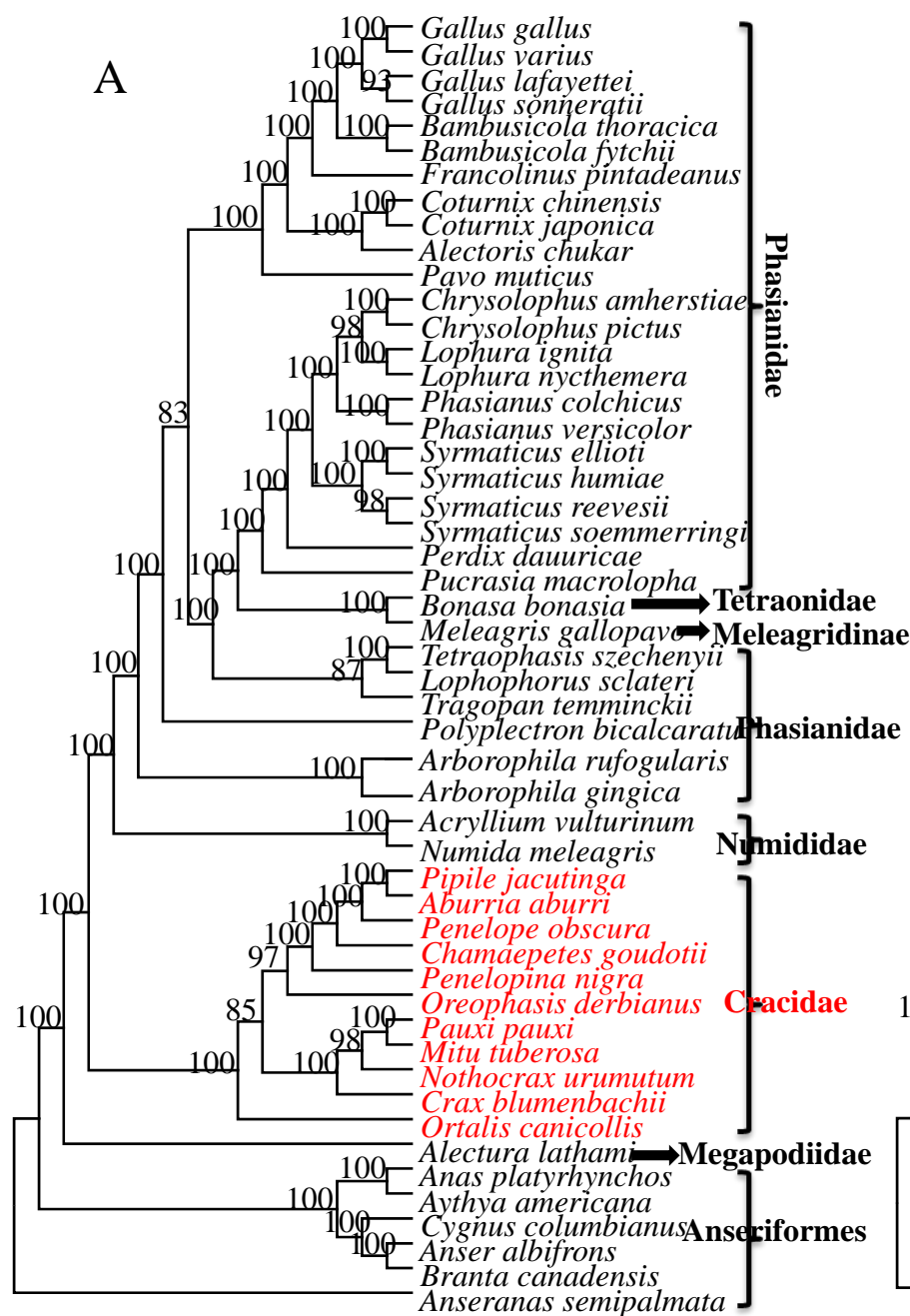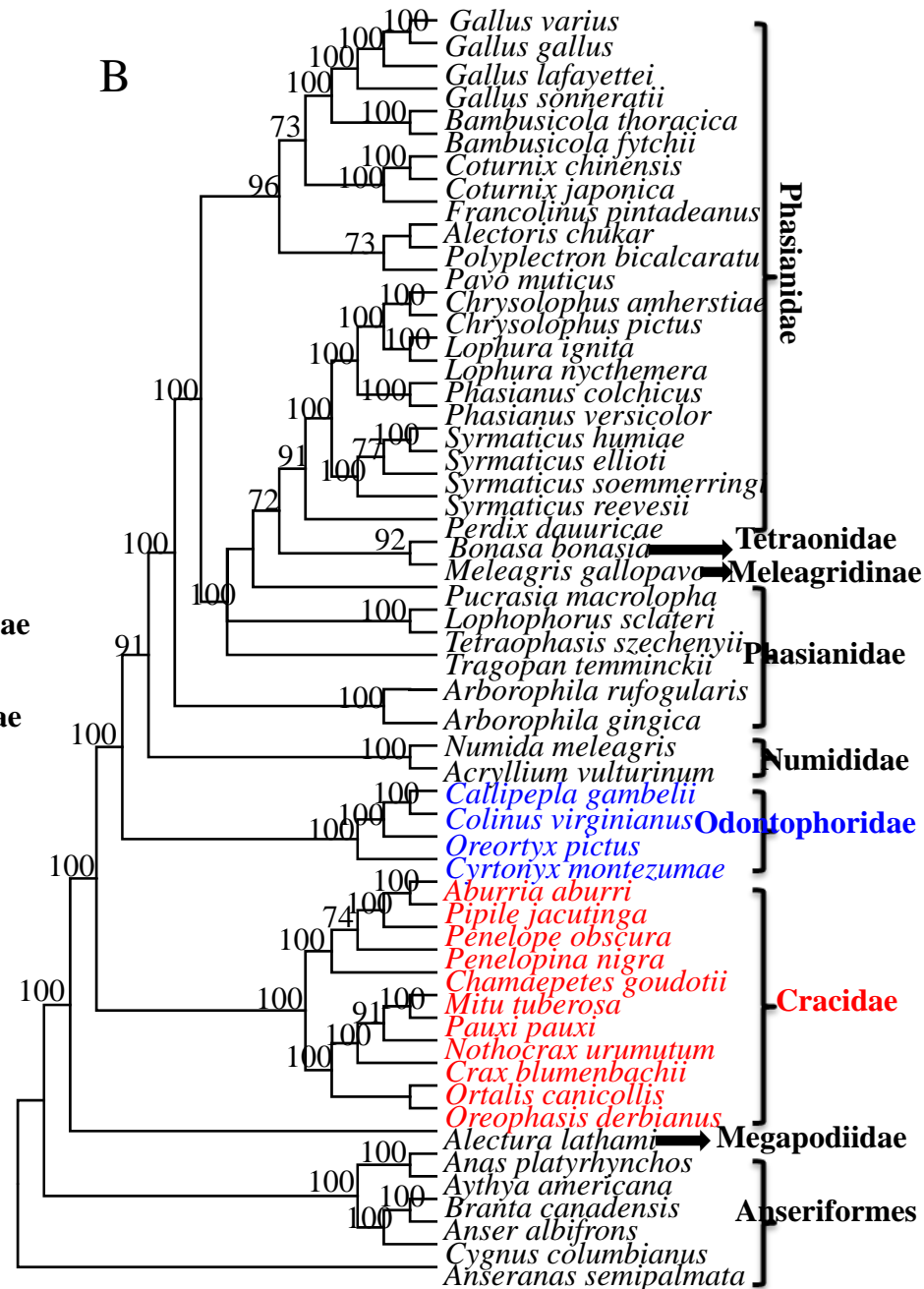

Supplement: Additional file 10 — Bayesian trees based on combined datasets of the Cracidae and Odontophoridae. Bayesian inference (BI) consensus trees based on combined datasets of the Cracidae and Odontophoridae. Anseriformes forms the outgroup. Bayesian posterior probabilities > 70% are indicated on the branches. (A) The BI tree for the dataset of 10,502 nucleotide positions for 11 species in the Cracidae (marked in red) and 40 galliform/anseriform birds that have complete mt genomes; (B) The BI tree for the dataset of 3,262 aligned nucleotide positions for 11 species in the Cracidae (marked in red), four in the Odontophoridae (marked in blue) and 40 galliform/anseriform birds. [file 1471-2148-10-132-S10.PDF]
