# Supplementary material for: A mitogenomic perspective on the ancient, rapid radiation in the Galliformes with an emphasis on the Phasianidae
Source: BMC Evol Biol. 2010 May 6;10:132. doi: 10.1186/1471-2148-10-132 (PMC2880301; doi:10.1186/1471-2148-10-132)
Supplement: Additional file 13 — Parameters of evolutionary models. Evolutionary models, log-likelihood values (-ln L), and settings identified by Modeltest for different DNA sequence datasets from the Galiformes. [file 1471-2148-10-132-S13.DOC]

Additional file 13:

Parameters of evolutionary models.

| Model parameter | *ND1* | *ND2* | *CoxI* | *CoxII* | *ATP8* | *ATP6* | *CoxIII* | *ND3* | *ND4* | *ND4L* | *ND5* | *CytB* | *ND6* | CR | *12S* | *16S* | rRNA | tRNA | 12 protein genes | 12 protein genes  (exclude 3^rd^ codon) | Mt genome | Cracid | NW quail |
| --- | --- | --- | --- | --- | --- | --- | --- | --- | --- | --- | --- | --- | --- | --- | --- | --- | --- | --- | --- | --- | --- | --- | --- |
| Model | GTR+I+G | GTR+I+G | TVM+I+G | TVM+I+G | GTR+I+G | GTR+I+G | TVM+I+G | TVM+I+G | GTR+I+G | TIM+I+G | TVM+I+G | TVM+I+G | K81uf+I+G | TVM+I+G | GTR+I+G | GTR+I+G | GTR+I+G | GTR+I+G | GTR+I+G | GTR+I+G | GTR+I+G | GTR+I+G | GTR+I+G |
| -ln L | 12370.2588 | 13577.2285 | 15871.6982 | 7210.8687 | 2251.8899 | 8520.6807 | 8232.0713 | 4441.3652 | 17183.2031 | 3447.3552 | 23613.7695 | 12522.9150 | 6636.7476 | 17927.4453 | 8845.7734 | 16266.3320 | 25167.8359 | 11302.9746 | 131103.7031 | 41385.1758 | 195293.6406 | 150252.0000 | 66571.3594 |
| I | 0.4375 | 0.3470 | 0.6074 | 0.5338 | 0.3727 | 0.4478 | 0.5490 | 0.4400 | 0.4152 | 0.4073 | 0.3813 | 0.5053 | 0.3453 | 0.1121 | 0.4405 | 0.4563 | 0.4593 | 0.5062 | 0.4635 | 0.6199 | 0.4490 | 0.4197 | 0.3901 |
| Gamma | 0.8878 | 0.6430 | 1.3135 | 0.7530 | 0.7711 | 0.7306 | 0.7797 | 1.0247 | 0.7425 | 0.6787 | 0.8862 | 0.8208 | 0.8026 | 0.5270 | 0.6284 | 0.7541 | 0.7409 | 0.6164 | 0.8085 | 0.6743 | 0.8932 | 0.8443 | 0.8023 |
| Base frequencies | | | | | | | | | | | | | | | | | | | |  |  |  |  |
| A | 0.3099 | 0.3502 | 0.3160 | 0.3514 | 0.3853 | 0.3267 | 0.3527 | 0.3057 | 0.3539 | 0.3352 | 0.3406 | 0.3312 | 0.1331 | 0.3038 | 0.3452 | 0.3607 | 0.3564 | 0.3370 | 0.3389 | 0.2412 | 0.3294 | 0.3459 | 0.3342 |
| C | 0.4053 | 0.4193 | 0.3884 | 0.3592 | 0.4091 | 0.4391 | 0.4094 | 0.4116 | 0.4210 | 0.4353 | 0.4217 | 0.4564 | 0.0694 | 0.2879 | 0.3065 | 0.3087 | 0.3081 | 0.2692 | 0.4178 | 0.3118 | 0.3625 | 0.3835 | 0.3733 |
| G | 0.0854 | 0.0607 | 0.0942 | 0.0953 | 0.0363 | 0.0552 | 0.0730 | 0.0956 | 0.0474 | 0.0618 | 0.0695 | 0.0630 | 0.3768 | 0.1139 | 0.1615 | 0.1579 | 0.1588 | 0.1583 | 0.0661 | 0.1562 | 0.1100 | 0.0793 | 0.0919 |
| T | 0.1994 | 0.1699 | 0.2014 | 0.1942 | 0.1692 | 0.1791 | 0.1649 | 0.1871 | 0.1777 | 0.1678 | 0.1682 | 0.1494 | 0.4208 | 0.2944 | 0.1868 | 0.1727 | 0.1767 | 0.2354 | 0.1772 | 0.2908 | 0.1981 | 0.1913 | 0.2006 |
| Rate matrix ([G<->T=1.00]) | | | | | |  |  |  |  |  |  |  |  |  |  |  |  |  |  |  |  |  |  |
| [A-C] | 0.5362 | 0.1280 | 1.3505 | 2.2112 | 0.0781 | 0.3140 | 303.1805 | 2.0099 | 0.1328 | 1.0000 | 0.7533 | 0.4732 | 1.0000 | 1.5184 | 7.6914 | 2.7993 | 3.9129 | 3.3009 | 0.4142 | 1.9547 | 0.6821 | 0.4265 | 0.4486 |
| [A-G] | 12.5606 | 5.7499 | 30.0664 | 57.8793 | 4.6741 | 14.4829 | 15719.7051 | 28.1245 | 14.1330 | 103.9321 | 17.6497 | 13.1469 | 33.5114 | 3.7991 | 36.2601 | 10.6390 | 16.0118 | 21.6552 | 14.8425 | 17.0456 | 6.9105 | 8.2207 | 5.3642 |
| [A-T] | 0.9788 | 0.3445 | 2.7163 | 5.0291 | 0.1044 | 0.6270 | 1795.5525 | 2.3540 | 0.6090 | 5.5651 | 1.8241 | 1.5806 | 2.4003 | 1.3436 | 7.4791 | 3.8038 | 4.6639 | 2.3178 | 1.0844 | 2.3636 | 1.1445 | 0.9227 | 0.8550 |
| [C-G] | 0.3327 | 0.1878 | 0.1233 | 1.7738 | 0.0004 | 0.2903 | 196.5067 | 0.5246 | 0.7823 | 5.5651 | 0.8557 | 0.3782 | 2.4003 | 0.8483 | 0.4717 | 0.2676 | 0.3570 | 0.4440 | 0.4595 | 0.8786 | 0.2029 | 0.3737 | 0.2980 |
| [C-T] | 8.9112 | 3.6089 | 30.0664 | 57.8793 | 1.4311 | 7.0487 | 15719.7051 | 28.1245 | 5.5009 | 39.1880 | 17.6497 | 13.1469 | 33.5114 | 3.7991 | 76.5988 | 38.2787 | 47.6774 | 28.7417 | 10.7288 | 14.6933 | 8.8096 | 6.8544 | 4.8523 |
| [G-T] | 1.0000 | 1.0000 | 1.0000 | 1.0000 | 1.0000 | 1.0000 | 1.0000 | 1.0000 | 1.0000 | 1.0000 | 1.0000 | 1.0000 | 1.0000 | 1.0000 | 1.0000 | 1.0000 | 1.0000 | 1.0000 | 1.0000 | 1.0000 | 1.0000 | 1.0000 | 1.0000 |
